# Whole Genome Sequencing of the Mutamouse Model Reveals Strain- and Colony-Level Variation, and Genomic Features of the Transgene Integration Site

**DOI:** 10.1038/s41598-019-50302-0

**Published:** 2019-09-24

**Authors:** Matthew J. Meier, Marc A. Beal, Andrew Schoenrock, Carole L. Yauk, Francesco Marchetti

**Affiliations:** 1 Environmental Health Science and Research Bureau, Health Canada, Ottawa, ON Canada; 2 Present Address: Ecotoxicology and Wildlife Health Division, Environment and Climate Change Canada, Ottawa, ON Canada; 3 Present Address: Existing Substances Risk Assessment Bureau, Health Canada, Ottawa, ON Canada

**Keywords:** Genome informatics, Genomics

## Abstract

The MutaMouse transgenic rodent model is widely used for assessing *in vivo* mutagenicity. Here, we report the characterization of MutaMouse’s whole genome sequence and its genetic variants compared to the C57BL/6 reference genome. High coverage (>50X) next-generation sequencing (NGS) of whole genomes from multiple MutaMouse animals from the Health Canada (HC) colony showed ~5 million SNVs per genome, ~20% of which are putatively novel. Sequencing of two animals from a geographically separated colony at Covance indicated that, over the course of 23 years, each colony accumulated 47,847 (HC) and 17,677 (Covance) non-parental homozygous single nucleotide variants. We found no novel nonsense or missense mutations that impair the MutaMouse response to genotoxic agents. Pairing sequencing data with array comparative genomic hybridization (aCGH) improved the accuracy and resolution of copy number variants (CNVs) calls and identified 300 genomic regions with CNVs. We also used long-read sequence technology (PacBio) to show that the transgene integration site involved a large deletion event with multiple inversions and rearrangements near a retrotransposon. The MutaMouse genome gives important genetic context to studies using this model, offers insight on the mechanisms of structural variant formation, and contributes a framework to analyze aCGH results alongside NGS data.

## Introduction

Mutagenicity assessment of chemicals and pharmaceuticals prior to commercial use is essential to the protection of public health and forms the primary component of human health risk assessment. The majority of mutagenicity assessment has been undertaken in genetically modified Salmonella bacteria (i.e., the Ames assay) providing a first-tier assay in conventional genotoxicity testing paradigms^1^. However, mutagenicity in non-metabolically competent bacterial lines, for many reasons, may not be recapitulated *in vivo* and reflect potential hazards for humans. Thus, most paradigms require follow-up testing in an *in vivo* model^2^ and the laboratory mouse has been an indispensable tool for understanding hazards posed by environmental chemicals and pharmaceuticals. Beginning in the late 1980s, transgenic rodent (TGR) mutation reporter models^3^ provided an unprecedented tool for quantifying mutations in mammalian cells. Since this time, TGR models have become the basis of *in vivo* mutagenicity assessment, and the use of TGR models to evaluate the safety of chemicals is described in the Organisation for Economic Co-operation and Development test guideline 488^4^.

The MutaMouse transgenic model is the most widely used TGR system in regulatory genetic toxicology^3,5^. The MutaMouse contains a recoverable *lacZ* transgene that can be isolated from the DNA of any tissue and used to score mutations with an *in vitro* positive selection assay^6,7^. The TGR assay provides unique opportunities to study mutations in any tissue. In addition, studies can be paired with next-generation sequencing to reveal details of changes in mutation spectrum to inform on the mechanism of action of the test agent^8,9^. Over 130 chemical and physical agents have been tested with the MutaMouse model (representing over 50% of the ~250 agents that have been tested in total)^3^. Given the importance of mutagenicity assessment and the prevalent use of the MutaMouse in such evaluations, the availability of a MutaMouse reference genome can provide a deeper understanding of its genetic traits that may be relevant to mutagenicity, chemical metabolism, and toxicology in general.

Analyses of gene expression profiles and other biochemical changes (e.g., enzyme activity) in response to chemicals exposure are increasingly used to complement genetic toxicology data in MutaMouse models^10–13^. Rapid advances in next-generation sequencing (NGS) technology have allowed the application of whole-genome approaches to understanding fundamental mechanisms of mutagenesis^14–17^. Fully annotated and characterized genomes of TGR models can improve the utility of these strains for genetic toxicology research by increasing the understanding of the potential biological consequences of genetic variants, and facilitating integration with modern genomic methods.

The high-quality genome assembly of the C57BL/6 mouse comprises the reference sequence against which all newly sequenced mouse genomes are compared^18^. The first studies using NGS to characterize mouse genomes^19–25^ established some important approaches to determine copy number variants^19,24^, genetic variation with respect to phenotype^23^ and structural variation among 17 mouse strains^20,22^. New methods for copy number variant (CNV) and structural variant (SV) discovery have been developed^16^ and analyses of repetitive elements on a genome-wide scale have improved our understanding of the role that this type of variation plays in disease^26^. Comparative transcriptomic studies have provided an initial survey of the molecular basis of phenotypic variation between inbred mouse strains^27^. Recently, the whole genome of C57BL/6J Eve, the female mouse from which the current C57BL/6J mice descend, has been reported and identified genetic variation that most closely represent recent generations of C57BL/6J mice than the original genome^28^.

The Mouse Genomes Project (http://www.sanger.ac.uk/science/data/mouse-genomes-project) has sequence data and variation for 36 fully sequenced strains as of 2019, while 16 of those also have completed *de novo* assemblies and gene annotation files^25^. Furthermore, the Mouse Genome Informatics database (MGI; http://www.informatics.jax.org/) provides access to a wide range of resources to facilitate studies on human health and disease^29^ to provide context to whole genome sequence data and integrate other data types and analytical tools (e.g., pathways, gene expression, phenotypes, etc.). This work has driven forward the field of comparative genomics in mice^30,31^. Finally, quantitative trait loci have become an important resource for linking genotype to phenotype^32^ and deep sequencing of multiple inbred mouse strains has been performed for such purposes^33^.

Here, we sequenced multiple MutaMouse animals with deep sequencing (>50X) and multiple platforms to obtain high-quality variant calls. We used array comparative genomic hybridization (aCGH) in parallel with NGS data to characterize putative CNVs. Finally, we compared the genetic variation between two geographically separated colonies of MutaMouse mice, to define the genetic variation existing within and across MutaMouse colonies, divergence from parental strains, and identify functional mutations. We also conducted the first characterization of the breakpoints at the transgene integration site.

## Results

### Sequence data

Seven MutaMouse animals, including five males obtained from a breeding colony maintained at Health Canada (HC) in Ottawa, Canada, and one male and one female obtained from a breeding colony at Covance, UK, were sequenced in this study. Sequencing on the Illumina HiSeq and NextSeq platforms together produced between 54 to 80X median genome-wide read depth for the five males from the HC colony. The NextSeq coverage for the animals from the Covance colony was 28–30X. Finally, the Pacific Biosciences reads from MutaMouse animal 2 aligned with a median depth of 3.5x. Mean fragment length, total raw basepairs, and median depth of aligned reads for all animals are shown in Table 1.

**Table 1:** Animals sequenced in this study and library/coverage statistics.

Animal		Platform and library type	Mean fragment length (bp)	Total raw bp	Median depth of aligned reads
Health Canada MutaMouse	1	Illumina TruSeq^a^	480	160,972,859,100	55X
	2	Illumina TruSeq and Nextera Mate Pair	3,078 (Nextera Mate Pair) 480 (TruSeq)	270,924,369,600	81X
		PacBio RSII^b^	20,000	11,900,000,000	3.5X
	3	Illumina TruSeq	450	159,512,702,850	55X
	4	Illumina TruSeq	450	159,691,600,650	54X
	5	Illumina TruSeq	444	196,932,569,400	71X
Covance MutaMouse	Male	Illumina Nextera Mate Pair	3,849 (Nextera mate pair)	144,615,033,150	32X
	Female	Illumina Nextera Mate Pair	3,414 (Nextera mate pair)	131,111,604,600	28X

^a^Libraries built by Génome Québec; sequencing carried out by Génome Québec and in-house at Health Canada.

^b^Sequencing and library construction performed by Génome Québec.

### Single nucleotide variants and inheritance from parental mouse strains DBA/2J and BALB/cJ

**Figure 1:**
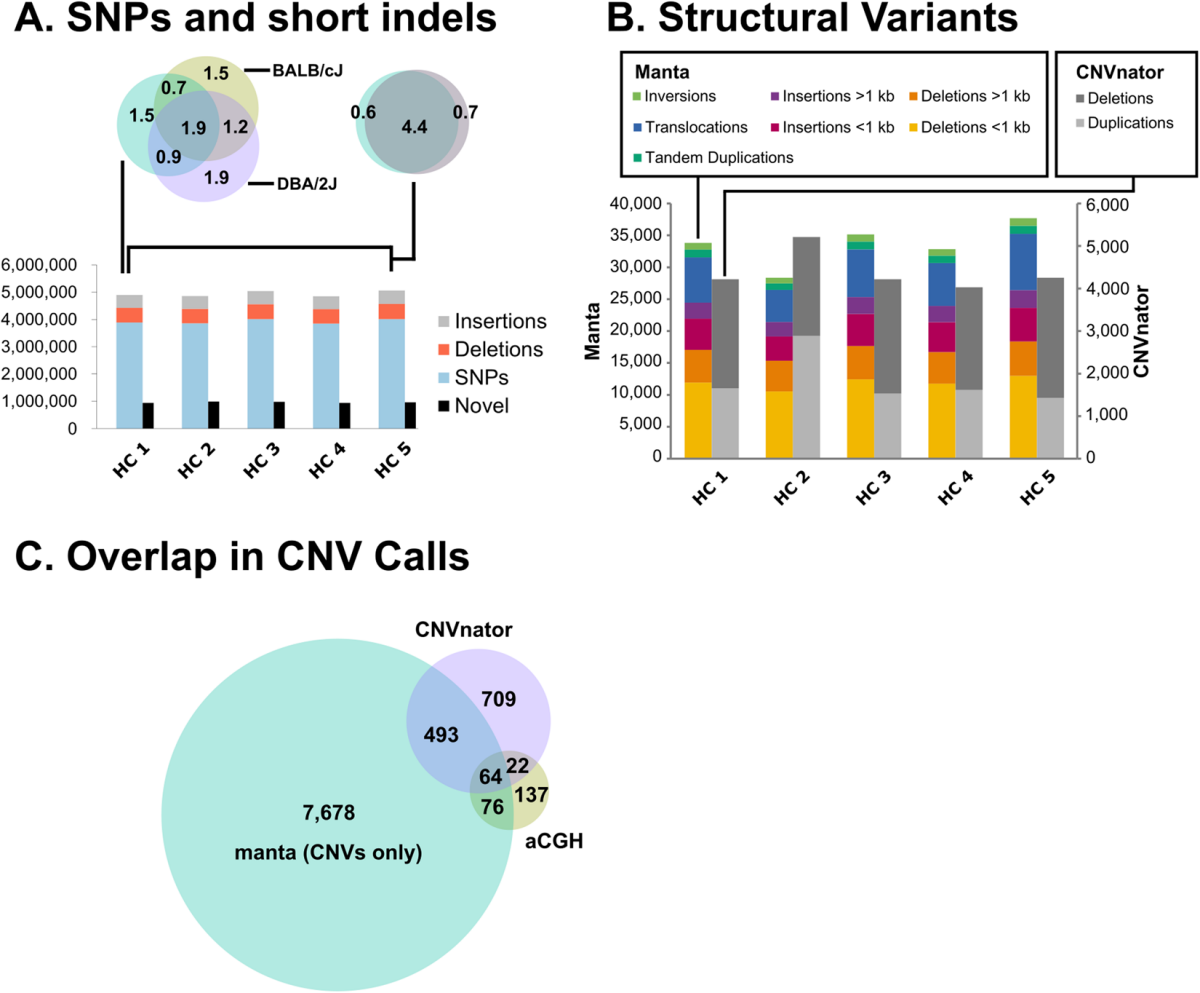
Summary of the number of genetic variants identified in MutaMouse animals from the Health Canada colony sequenced for this study. (**A**) The number of small variants discovered in each animal and the relative proportions of SNVs, insertions, and deletions. The number of novel variants (i.e., those not found in dbSNP v142) is shown as a black bar. A consistent number of small variants was observed between animals, with an average pairwise overlap of 85%. A Venn diagram with the number of variants (in millions) shared by two representative MutaMouse individuals is shown above right, and a Venn diagram showing the overlap with parental strains DBA/2 and BALB/c is shown above left. (**B**) Structural variants (SVs) identified in MutaMouse using NGS data and two different algorithms (Manta and CNVnator). Nearly 10-fold more SVs were predicted by Manta, which uses paired-end information to determine discordant reads. CNVnator was used to predict the locations of copy number variants greater than 250 bp in length. (**C**) The overlap in CNV calls between different methods. This analysis was limited to CNVs only found in all five animals. The majority of each call set does not overlap with the others, reinforcing the idea that multiple algorithms and methods should be used to call structural variants.

**Figure 2:**
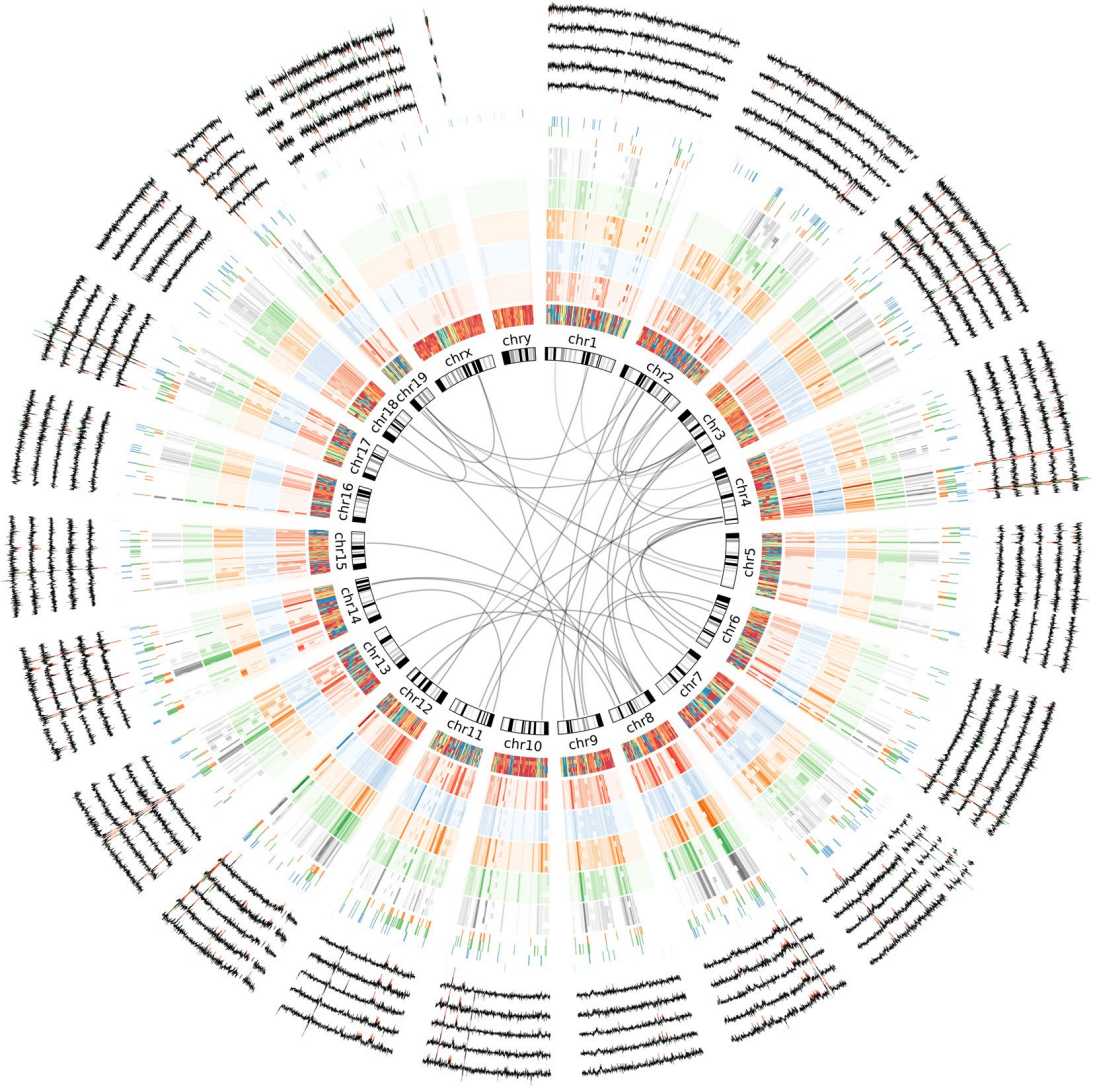
Circos plot^54^ showing genetic variation between five individual MutaMouse animals from a colony maintained at Health Canada. Links represent structural translocations identified in all mice sequenced. Each outside track has 5 individual components, one for each mouse sequenced (MutaMouse HC 1, 2, 3, 4, 5, from inside to out). The tracks from inside to outside represent (1) runs of homozygosity (where blue is homozygous and red is heterozygous – immediately adjacent to chromosome numbering), (2) Red track: SNV density per megabase of DNA sequence for all SNVs, (3) Blue track: SNV density for novel SNVs, (4) Orange track: SNV density for variants inherited from DBA/2J exclusively, (5) Green track: SNV density for variants inherited from BALB/cJ exclusively, and (6) Grey track: SNV density for variants inherited from both parents. The next tracks show copy number variants for the parental strains DBA/2J and BALB/cJ (orange and green, respectively), followed by copy number variants in MutaMouse predicted by CNVnator (blue). Finally, the line plots show the log2 values for aCGH probes averaged over 1 megabase intervals, with red highlights showing deleted segments and green highlights showing duplicated segments for each mouse.

We predicted single nucleotide variants (SNVs) and short insertions or deletions (indels) using the HaplotypeCaller from GATK, relative to the mm10 reference genome (Genome Reference Consortium Mouse Build 38). Raw variants were filtered using variant quality score recalibration with a sensitivity of 99.9%. In total, the GATK workflow predicted between 4,949,501 to 5,161,277 variants for each animal (Fig. 1A; Supplemental Table S1). The majority of variants were SNVs (78–80%), and most (80–84%) were already present in dbSNP v142. We found that many of these SNVs (38% of MutaMouse SNVs) were present in both parental strains (Fig. 1A), and some variants were inherited only from BALB/cJ (13%) or DBA/2J (18%); in total, 69% of all MutaMouse SNVs appear to originate from a parental strain. Here, we define BALB/cJ and DBA/2J as the parental strains, although the MutaMouse was created from the mating of two [BALB/cJ X DBA/2J]F_1_ hybrids. It is likely that random assortment during the creation of the MutaMouse strain resulted in a slight bias toward DBA/2J chromosomal segments in the final strain 40.6. In particular, there is an obvious lack of BALB/cJ sequences in the p arms of chromosomes 2 and X (Fig. 2). Of the remaining fraction of MutaMouse SNVs (31%) not found in parental strains, 11% (i.e., approximately 1/3 of the non-parental MutaMouse SNVs) are found in dbSNP. This 11% portion could represent: (1) false negatives in the BALB/cJ or DBA/2J reference sequences used; (2) variants that are found in BALB/c or DBA/2J but not in those animals sequenced by the mouse genomes project; and, (3) false positives resulting from systematic or bioinformatic errors that are in both dbSNP and the MutaMouse data. The remaining 20% of variants (i.e., approx. 937,000–989,000, depending on the animal; Supplemental Table S1) identified in this study are putatively private MutaMouse variants that arose during the over 25 years of breeding at HC.

### Structural and copy number variants

Both aCGH and NGS data were used to identify SVs and CNVs. There were 300 CNVs called using R-Gada from the aCGH probe values, of which 297 could be converted to mm10 coordinates. NGS data was analyzed using two methodologies: Manta and CNVnator. Manta was used to call putative SVs (translocations, tandem duplications, deletions, and insertions) from Illumina NGS data based on discordant read pairs and local *de novo* assembly (Figs 1B, 2; Supplemental Table S1). Each MutaMouse animal had an average of approximately 17,000 deletions, 7,000 insertions, 1,100 tandem duplications, 1,000 inversions, and 7,000 translocation breakpoints. When considering only SV calls that were common to all animals, there were 4,795 deletions, 1,961 insertions, 190 tandem duplications, 239 inversions, and 686 translocation breakpoints. CNVnator, which predicts CNVs from NGS data based on read depth, identified an average of approximately 2,500 deletions and 1,800 duplications per animal.

Because SVs calls are less reliable than SNV calls, we limited our dataset to predicted variants found in all five animals sequenced to enrich for robust predictions and determined the overlap in predicted CNVs among aCGH, CNVnator, and Manta (Fig. 1C). In total, 64 events were predicted by all three methods (Fig. 1C). This number mainly reflects the larger CNVs that were predicted confidently by aCGH. However, each method produced a large number of predicted SVs that are exclusive to that dataset. Because our analysis included only SVs that were found in all five animals, many spurious calls were eliminated; thus, the significant differences between call sets produced by aCGH, CNVnator, and Manta can be attributed largely to analytical differences between the methods rather than false positives. Overall, these methods provide a picture of the genome that encompasses many different SV types across different scales.

### Biological consequences of genetic variants

**Figure 3:**
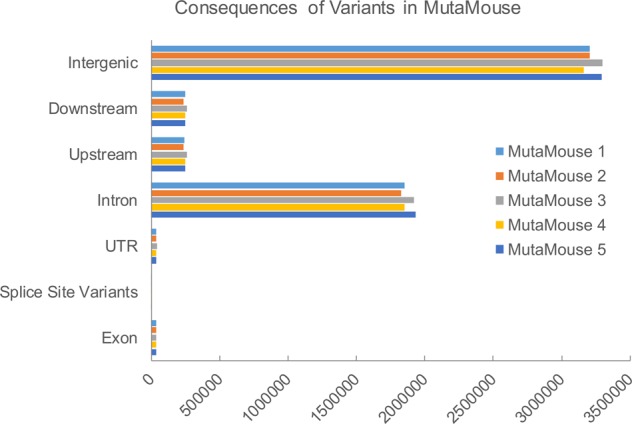
The number of predicted locations of variants sequenced in five MutaMouse animals. The majority of variants are found in intergenic regions, introns, or upstream and downstream non-coding sequences. Only 0.62% of variants (an average of 35,000 per animal) are found within exons.

We used the Ensembl Variant Effect Predictor (VEP) and SnpEff to characterize the predicted biological effects of the SNVs and small indels identified through sequencing. Most of these variants fall into the category of intergenic or intronic (average of 3.2 million and 1.9 million, respectively; Fig. 3). Many variants also fall within 5 kb upstream or downstream of genes (average of 0.25 million each). Comparatively few variants fall within exons (~35,000), 3′ UTRs (~33,000), 5′ UTRs (~5,400), splice sites (~3,700), or otherwise influence transcription (~12). Of variants that fall within exons, an average of 10,045 missense variants were observed in each mouse (9,520–10,558), while synonymous variants representing between 15,833 and 18,032 of the total variants observed. No nonsense or frameshift mutations were identified.

We focused our analysis of variants on those that were previously unreported and not inherited from the parental mouse strains, since these are the candidate variants that may alter the physiological state of the MutaMouse model compared to other mouse models. We constrained our analysis to variants that were present in genic regions from pathways that are relevant to genetic toxicology studies: genes involved in cancer progression, cell cycle, DNA repair, or xenobiotic metabolism (Table 2, Supplemental Table 2). When considering small variants (SNVs and indels) private to MutaMouse and identified as potentially deleterious, very few variants emerge: 26 for cancer genes, 32 for cell cycle genes, 5 for DNA repair genes, and 1 for xenobiotic metabolism. Even fewer of these variants were found across all the animals sequenced (Table 2). Overall, most of the identified variants already exist in other mouse strains. Furthermore, most variants do not affect coding sequences, and instead lie in regulatory regions (Fig. 3).

**Table 2:** Variants (SNVs and short indels) identified in MutaMouse in the genes from pathways that are important for genetic toxicology studies.

Animals	Cancer Progression^a^ (642 genes)			Cell Cycle^b^ (1,434 genes)			DNA repair^b^ (408 genes)			Xenobiotic Metabolism^b^ (45 genes)		
	Variants within genes	Non synonymous	SIFT score deleterious^c^	Variants within genes	Non synonymous	SIFT score deleterious^d^	Variants within genes	Non synonymous	SIFT score deleterious^c^	Variants within genes	Non synonymous	SIFT score deleterious^c^
MutaMouse 1	93,708 (15,099)^c^	301 (34)	52 (8)	133,383 (21,452)	659 (47)	98 (23)	35,592 (5,567)	244 (3)	27 (0)	3,483 (630)	13 (1)	2 (1)
MutaMouse 2	80,922 (14,455)	313 (38)	54 (10)	135,318 (21,943)	598 (47)	96 (24)	34,416 (5,475)	222 (9)	27 (4)	3,295 (586)	11 (1)	2 (1)
MutaMouse 3	98,330 (15,681)	354 (45)	67 (20)	147,108 (23,716)	745 (58)	117 (29)	39,493 (6,055)	246 (3)	27 (1)	3,469 (643)	11 (1)	2 (1)
MutaMouse 4	89,172 (14,906)	324 (44)	65 (20)	136,668 (21,821)	621 (51)	102 (26)	37,618 (5,802)	238 (7)	30 (3)	3,326 (583)	11 (1)	2 (1)
MutaMouse 5	110,565 (16,748)	327 (39)	56 (11)	138, 297 (22,785)	645 (49)	98 (23)	39,869 (6,156)	258 (5)	28 (1)	3,278 (592)	11 (1)	2 (1)
MutaMouse Covance Female	71,057 (9,512)	2,379 (37)	42 (20)	111,611 (14,304)	541 (31)	91 (17)	28,344 (3,663)	198 (6)	29 (3)	2,932 (362)	10 (1)	2 (1)
MutaMouse Covance Male	71,513 (9,820)	242 (44)	43 (22)	112,389 (14,734)	545 (36)	90 (17)	28,474 (3,478)	199 (7)	29 (3)	2,934 (354)	10 (1)	2 (1)
Common	55,389 (7,682)	219 (22)	36 (5)	95,155 (12,129)	479 (35)	68 (18)	27,121 (3,309)	184 (2)	19 (0)	2,979 (358)	11 (1)	2 (1)
Total	132,221 (24,470)	407 (66)	71 (26)	180,472 (34,966)	870 (74)	129 (32)	46,554 (8,808)	280 (11)	34 (5)	3,938 (904)	13 (1)	2 (1)

^a^COSMIC cancer genes (Mouse homologs).

^b^Retrieved using BioMart.

^c^SIFT scores calculated using Ensembl Variant Effect Predictor; only canonical transcripts reported (i.e., only one consequence is reported per variant).

^d^Not reported in dbSNP 142.

^e^Subset of variants that are present in all mice.

^f^Total count of variants (i.e., present in 1 or more mouse).

Within the genes of interest, we identified 22 genes that possess SNVs unique to MutaMouse that are also predicted to deleteriously affect the function of a gene product, as indicated by a SIFT score of <0.05^34^ (SIFT was run as part of the VEP analysis; Table 3). Some of these genes (e.g., *Rrs1*, *Hsp90ab1*) possess multiple variants within their coding sequences, while others (e.g., *Ahr*) possess only one. *Ahr* is the only xenobiotic metabolizing gene that we identified containing a variant with a deleterious SIFT score (chr12:35,508,182 T > C, which alters the codon Atc > Gtc resulting in an I > V amino acid change within exon 8). This variant was homozygous in every MutaMouse animal sequenced. We also found two DNA repair genes, *Rev1* and *Cdc5l*, with variants that result in amino acid substitution: chr1:38,088,013 T > C, chr1:38088020 C > G (K > E and K > N amino acid changes) in *Rev1*; and chr17:45,407,947 G > A (causing a T > M amino acid change) in *Cdc5l*. The remaining genes that possessed detrimental variants were involved with cell cycle regulation or cancer progression. We expected this result because these pathways contain 1,434 and 642 genes, respectively, which is more than DNA repair, with 408 genes, and xenobiotic metabolism, with 45 genes. Overall, although the SIFT algorithm predicted that some of these variants are deleterious, we have yet to discover phenotypic evidence that any of these pathways are significantly affected in MutaMouse.

**Table 3:** Genes in MutaMouse affected by potentially deleterious single nucleotide variants (as determined by SIFT) that are relevant to genetic toxicology studies.

Gene Symbol	Number of SNVs in gene for each animal					Chromosomal position(s) and nucleotide change(s)	Amino acid change (codon change)	Protein^a^	Functions
	HC 1	HC 2	HC 3	HC 4	HC 5				
Ahr	1	1	1	1	1	chr12:35508182 T > C	I/V (Atc/Gtc)	Aryl hydrocarbon receptor	Xenobiotic metabolism, Cell cycle
Arpp19	1	1	1	1	1	chr9:75056711 C > G	P/R (cCg/cGg)	cAMP-regulated phosphoprotein 19	Cell cycle
Cbl	0	0	0	1	2	chr9:44151504 C > T, chr9:44151527 A > T	A/T (Gct/Act), I/N (aTt/aAt)	E3 ubiquitin-protein ligase CBL	Cancer progression
Ccnb2	0	0	0	1	0	chr9:70410215 G > A	T/M (aCg/aTg)	G2/mitotic-specific cyclin-B2	Cell cycle
Ccnb3	1	1	1	1	1	chrX:7025679 T > A	N/I (aAc/aTc)	G2/mitotic-specific cyclin-B3	Cell cycle
Cdc5l	0	0	1	0	0	chr17:45407947 G > A,	T/M (aCg/aTg)	Cell division cycle 5-like protein	DNA repair, Cell cycle
Cdk4	0	0	1	0	1	chr10:127064302 G > A	A/T (Gcc/Acc)	Cyclin-dependent kinase 4	Cancer progression, Cell cycle
Cenpt	0	0	0	1	0	chr8:105845366 G > A	R/C (Cgc/Tgc)	Centromere protein T	Cell cycle
Eif4a2	0	0	2	2	0	chr16:23113181 G > T, chr16:23113194 G > T	G/V (gGt/gTt), R/S (agG/agT)	Eukaryotic initiation factor 4A-II	Cancer progression
Hsp90ab1	0	1	9	9	1	chr17:45568279 T > A, chr17:45568347 T > G, chr17:45568434 C > T, chr17:45568456 T > C, chr17:45568465 G > A, chr17:45568474 T > G, chr17:45568484 G > A, chr17:45568493 T > C, chr17:45568520 C > T, chr17:45568531 G > T, chr17:45568532 G > A	D/V (gAt/gTt), K/N (aaA/aaC), M/I (atG/atA), D/G (gAc/gGc), A/V (gCa/gTa), K/T (aAg/aCg), R/W (Cgg/Tgg), N/D (Aac/Gac), V/M (Gtg/Atg), P/H (cCc/cAc), P/S (Ccc/Tcc)	Heat shock protein HSP 90-beta	Cancer progression
Mapk6	1	1	1	1	1	chr9:75388680 T > A	Q/L (cAg/cTg)	Mitogen-activated protein kinase 6	Cell cycle
Mplkip	2	2	2	2	2	chr13:17695605 C > T, chr13:17695681 C > A	R/W (Cgg/Tgg), P/Q (cCg/cAg)	M-phase-specific PLK1-interacting protein	Cell cycle
Msi2	1	1	1	1	1	chr11:88687461 C > A	V/F (Gtc/Ttc)	RNA-binding protein Musashi homolog	Cancer progression
Nabp2	0	1	0	0	1	chr10:128408557 C > T	G/S (Ggc/Agc)	SOSS complex subunit B1	DNA repair, Cell cycle
Nanog	1	0	1	1	0	chr6:122707814 T > G	L/W (tTg/tGg)	Homeobox protein	Cell cycle
Nek1	1	1	1	1	1	chr8:61054575 C > A	R/S (Cgt/Agt)	Serine/threonine-protein kinase	Cell cycle
Rev1	0	2	0	2	0	chr1:38088013 T > C, chr1:38088020 C > G	K/E (Aaa/Gaa), K/N (aaG/aaC)	DNA repair protein REV1	DNA repair
Rprd1b	0	1	0	0	0	chr2:158047932 G > T	K/N (aaG/aaT)	Regulation of nuclear pre-mRNA domain-containing protein 1B	Cell cycle
Rrs1	13	12	13	13	13	chr1:9545597 C > T, chr1:9545621 A > T, chr1:9545666 C > T, chr1:9545674 C > T, chr1:9545762 C > A, chr1:9545801 C > A, chr1:9545860 C > A, chr1:9545917 G > C, chr1:9545925 C > A, chr1:9546313 G > A, chr1:9546338 T > A, chr1:9546341 G > T, chr1:9546377 C > T	T/M (aCg/aTg), E/V (gAg/gTg), T/I (aCc/aTc), R/C (Cgc/Tgc), P/Q (cCg/cAg), P/Q (cCg/cAg), R/S (Cgc/Agc), V/L (Gtg/Ctg), D/E (gaC/gaA), E/K (Gag/Aag), L/H (cTt/cAt), R/L (cGa/cTa), T/M (aCg/aTg)	Ribosome biogenesis regulatory protein homolog	Cell cycle
Tdpoz2	1	1	1	1	1	chr3:93652273 G > C	L/V (Ctc/Gtc)	TD and POZ domain-containing protein 2	Cancer progression
Thrap3	3	1	3	3	3	chr4:126165462 T > C, chr4:126165536 G > A, chr4:126165542 G > A	I/M (atA/atG), R/W (Cgg/Tgg), R/W (Cgg/Tgg)	Thyroid hormone receptor-associated protein 3	Cancer progression
Tjp3	0	0	1	0	0	chr10:81274478 T > G	(Atc/Ctc)	Tight junction protein ZO-3	Cell cycle
Total	29	32	46	45	32				

^a^Panther protein family/subfamily or class.

Of the 1,094 and 1,842 deletions that were found in all the mice using Manta and CNVnator (the NGS-based methods for SV calling), respectively, 13 deletions (Table 4) occurred in genes of toxicological interest. We predict that these deletions do not have a significant impact on protein function since they all fall in intergenic (upstream or downstream) or intronic regions. However, they may impact expression or transcript processing of the genes since regulatory regions and introns are deleted.

**Table 4:** Genes in MutaMouse affected by potentially detrimental deletions discovered with both CNVnator and Manta (NGS-based tools) that are relevant to genetic toxicology studies.

Gene	Predicted effects of variant	Chromosomal coordinates^a^	Number of deleted bases	Protein	Functions	Copy numbers observed in MutaMouse population
Grk5	intron variant	chr19:60925302–60934033	8,731	G protein-coupled receptor kinase 5	Cell cycle	0 or 1
Kif5b	downstream gene variant	chr18:6196144–6199729	3,585	Kinesin-1 heavy chain	Cancer progression	0 or 1
Cdh11	intron variant	chr8:102660453–102663326	2,873	Cadherin-11	Cancer progression	0
Tnks	intron variant	chr8:34882786–34885003	2,217	Tankyrase-1	Cell cycle	0
Dctn6	downstream gene variant	chr8:34087155–34089063	1,908	Dynactin subunit 6	Cell cycle	0
Nrg1	intron variant	chr8:31969110–31970943	1,833	Neuregulin 1	Cancer progression	0
Ncoa2	upstream gene variant	chr1:13378446–13380234	1,788	Nuclear receptor coactivator 2	Cancer progression	0
Arhgap26	intron variant, non-coding transcript variant	chr18:38663457–38665186	1,729	Rho GTPase-activating protein 26	Cancer progression	0 or 1
Rec114	intron variant	chr9:58685759–58687011	1,252	Meiotic recombination protein REC114	Cell cycle	0 or 1
Wdr62	intron variant	chr7:30275960–30276784	824	WD repeat-containing protein 62	Cell cycle	0
Prkdc	intron variant	chr16:15638729–15639323	594	DNA-dependent protein kinase catalytic subunit	Cell cycle, DNA repair	0
Kmt2e	intron variant	chr5:23494281–23494730	449	Histone-lysine N-methyltransferase 2E	Cell cycle	0
Fancc	intron variant	chr13:63480021–63480458	437	Fanconi anemia group C protein homolog	Cancer progression, DNA repair	0

^a^Coordinates reported by Manta are shown since they allow nucleotide resolution, while CNVnator reports only 250 bp bin locations.

**Figure 4:**
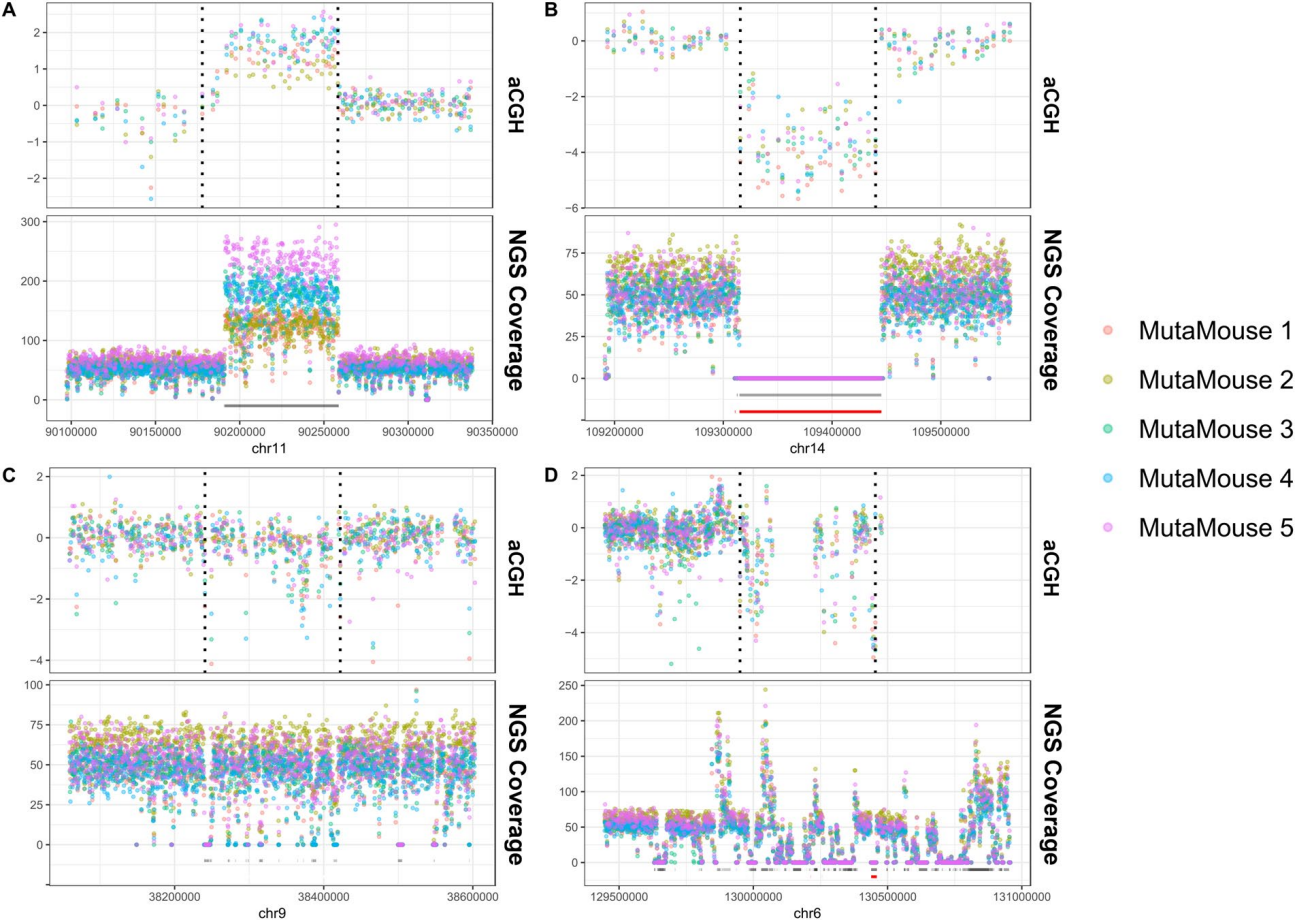
Four examples of CNVs predicted using aCGH (probes shown as points in top panels; CNV call between dotted lines) overlaid with their respective NGS coverage (bottom panels; NGS coverage is shown as points; CNVnator calls from NGS data shown as black lines below coverage; Manta calls from NGS data shown as red lines below coverage). We find that some CNVs predicted using aCGH are consistent across the entire call region when compared with NGS coverage. On the other hand, many regions predicted as CNVs by aCGH represent regions of the genome encompassing many small deletions and/or gains. (**A**) A duplication on chromosome 11 showing congruent results between aCGH and NGS. (**B**) A deletion on chromosome 14 showing congruent results between aCGH and NGS. (**C**) A deletion on chromosome 9 predicted as one large deletion by aCGH that NGS has revealed is composed of many small deletions. (**D**) A deletion on chromosome 6 predicted as one large deletion by aCGH that NGS revealed is composed of many small deletions as well as several copy number gains.

There is little overlap between NGS-based and aCGH CNV calls because the probe spacing for aCGH (on the order of kilobases) is much coarser than the resolution of NGS-based methods; thus, CNVnator is able to predict much smaller CNVs. Each of these methods is expected to provide complementary information to access different resolutions of SVs. To increase the reliability of our aCGH calls, we overlaid NGS coverage with each CNV call made using aCGH data. This revealed some instances where calls interpreted by the R-gada algorithm as one large event were actually composed of several small events (Fig. 4). Supplemental Table S3 describes the CNV calls made using aCGH and the CNV classification type (simple vs. complex) that we established using NGS coverage data to eliminate regions that R-gada predicted as CNVs. We classified events as complex SVs (encompassing multiple insertions and deletions) if they contained more than one CNV called by CNVnator. After this analysis, we classified 142 of the R-gada calls as simple and 156 as complex.

### Transgene integration site

**Figure 5:**
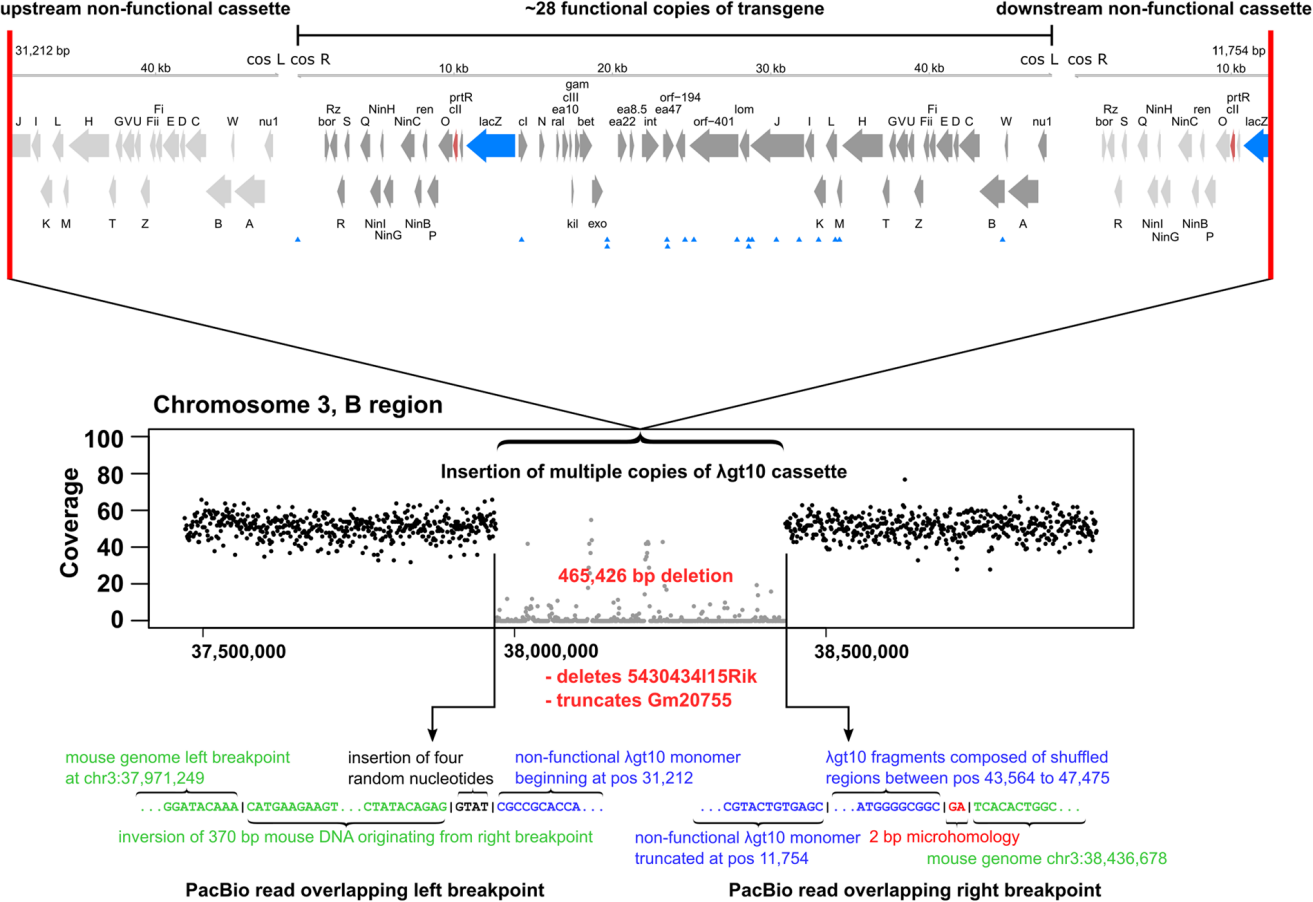
Assembly of λgt10-*lacZ* transgene integration site using PacBio read data and NGS coverage data. There are approximately 29 copies of the λgt10 ori replication site^35^. However, we show here that at least one copy (at the right breakpoint, downstream of the integration site) is non-functional. There are 18 SNVs that are constitutional in every copy of the transgene (shown by blue arrows in the top panel for one transgene monomer, and listed in Table 5). The breakpoints near the integration site were resolved by the use of long PacBio reads that map to both the λgt10 transgene sequence and mouse genomic sequence (bottom panel, colored text). The integration of the transgene comprised an unusual event involving (1) the deletion of ~465 Kbp of mouse genomic sequence, (2) the inversion of 370 bp of mouse genomic sequence from the downstream breakpoint to the upstream breakpoint, (3) the insertion of two partial truncated transgene monomers at either breakpoint, and (4) the insertion of shuffled transgene sequence at the downstream breakpoint as well as the insertion of several random nucleotides at the upstream breakpoint.

The transgene integration site is on the B region of chromosome 3^35,36^, but the precise breakpoints are unknown. We identified three long (PacBio) reads that overlapped the breakpoints, containing both mouse and transgene sequence. Surprisingly, the breakpoints were found at either end of a 465,426 bp homozygous deletion, accompanied by a 370 bp inversion detected by discordant mate pairs and identified by Manta (a deletion spanning chr3:37,971,249–38,436,675, with the inversion of sequence from chr3:38,436,306–38,436,675 joined to the left breakpoint; Fig. 5). In the mm10 reference genome, there is a long terminal repeat (RMER20A#LTR/ERVK) present upstream of this locus. The deletion observed in MutaMouse is not present in either of the parental mouse strains, and therefore likely occurred concurrently with the integration of the transgene. We have observed this event in all MutaMouse animals analyzed to date, from both the HC and Covance colonies.

The complete sequence of the transgene in MutaMouse, as well as the precise breakpoints of insertion, were hitherto unknown. *De novo* assembly of NGS mate pair reads that were collected by first aligning them to a manually-assembled reference of λgt10^35^ revealed several constitutional SNVs and short indels that are found in every copy of the transgene in MutaMouse (Fig. 5; Table 5). These variants do not alter the protein coding sequences in the *lacZ* gene region and the sequence of the transgene is otherwise identical to the manually assembled version.

**Table 5:** Constitutional variants that are present in all copies of the MutaMouse transgene across all animals sequenced.

Position	Reference Base	Alternate Base
137	AG	A
14266	C	CG
19670	T	A
19673	A	C
23445	G	C
23481	A	G
24583	TA	T
25143	A	G
27867	T	C
28598	G	A
28599	T	A
28817	A	T
30349	CT	C
31786	A	G
33016	T	C
34070	A	G
34331	T	C
44629	T	C,A,G

### Genetic similarity between two separate lineages of mutaMouse

**Figure 6:**
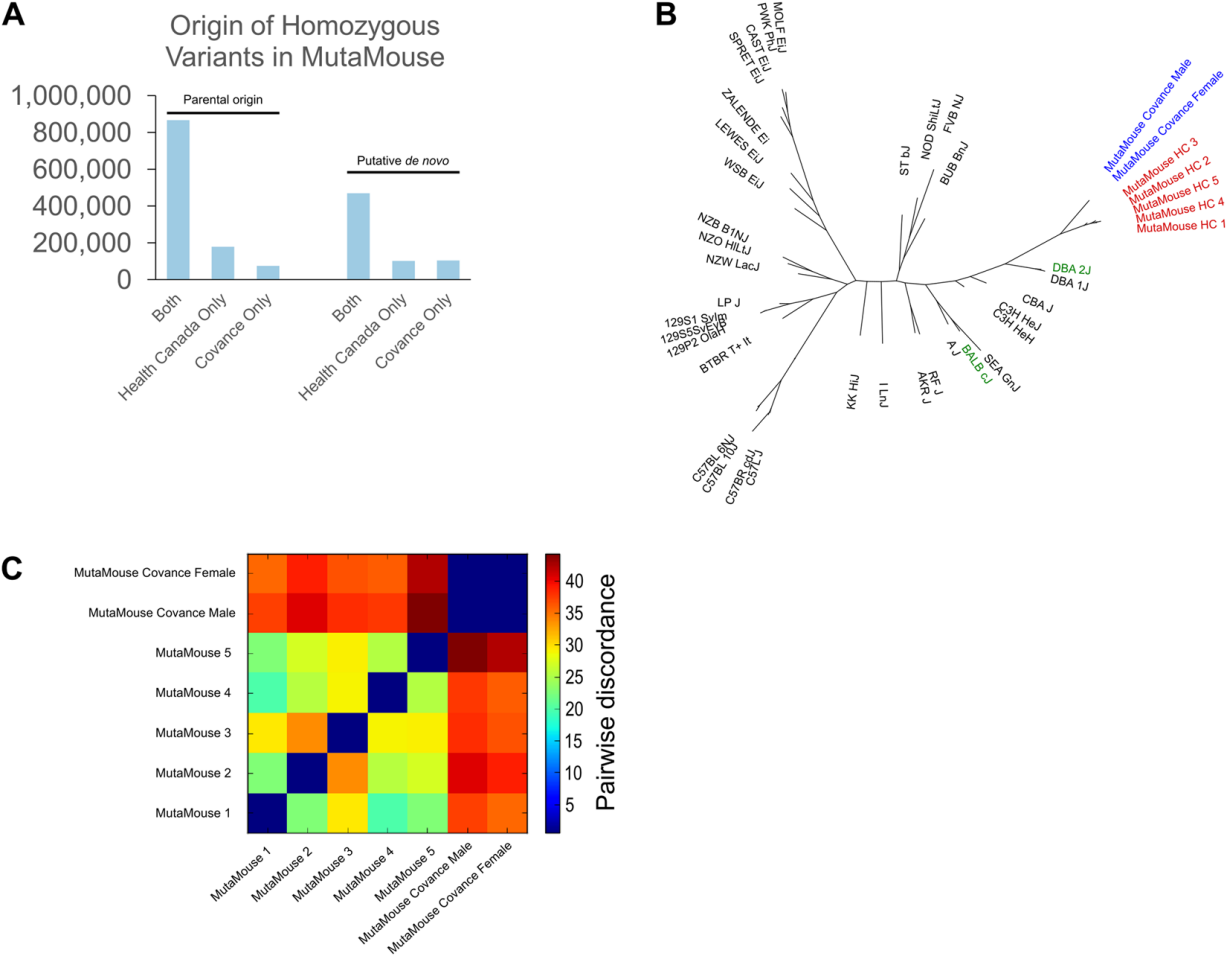
Comparison of variants discovered in MutaMouse colonies that were geographically isolated for 23 years of breeding (78 generations). (**A**) More SNVs of parental origin were retained in the Health Canada colony than the Covance colony, which experienced a population bottleneck circa 2006. The number of putative *de novo* SNVs is comparable, suggesting that the germline mutation rates do not differ between the populations. (**B**) On a phylogenetic tree, the MutaMouse colony from Covance forms a distinct branch, which is consistent with our finding that (**C**) Animals from the Covance MutaMouse colony have higher genotype discordance with animals from Health Canada than with each other.

To explore the genetic differences between the MutaMouse animals from the HC and Covance colonies, we compared the number of homozygous SNVs that were also found in the parental mice (Fig. 6A, left) or not (Fig. 6A, right), broken down further into which colony the SNV was present (both, HC only, or Covance only). The Covance colony retained marginally fewer parental SNVs than the HC colony (942,713 vs 1,046,884 in total). The number of non-parental SNVs was comparable between the two colonies (570,600 and 574,935). In total, when considering SNVs that were non-parental and homozygous, the HC and Covance colonies shared 181,821 SNVs, while 47,847 non-parental SNVs segregated in the HC colony and 17,677 segregated in the Covance colony. Furthermore, the two colonies segregate into separate branches on a phylogenetic tree based on their SNVs (Fig. 6B). Based on pairwise genotype concordance (Fig. 6C), mice originating from the two colonies are highly discordant (i.e., calculated as [# of different sites]/[total sites]; discordance values range between 35.5 and 44.3 for animals between colonies). The two mice within the Covance colony are more similar to one another (discordance of 0.53), while mice from the HC colony show more discordance within their own group (discordance between 19.9 and 33.7).

To exclude that these results were due to differences in the median genome-wide read depth between the two colonies (54–81X for the HC colony, 28–32X for the Covance colony) we downsampled the sequenced data for the five HC mice to an average depth of 28–30X and found that there was no significant difference in the number of variants that were called (Supplemental Table S4). Similarly, there was no impact of downsampling the data from HC MutaMouse 2 generated with the same sequencing platform as the Covance mice (Supplemental Table S5).

## Discussion

We generated the whole genome sequence of the MutaMouse model and characterized variants in multiple individuals from two geographically isolated colonies using a high sequence depth combined with aCGH. Analysis of these variants identified novel coding variants (mainly SNVs) that may influence genes involved in pathways related to toxicological responses, which is the primary use of this mouse model. No SVs that affected the coding sequence of these genes were identified, however, there were many SVs within the intronic, upstream, and downstream sequences that may modulate gene function. Characterization of the transgene integration site showed that it does not interrupt any genes. Finally, our work provides a reference sequence for future genomic studies on this important toxicological model.

A novel aspect of our investigation is the characterization of genetic variability between two MutaMouse colonies. Previous work has focused on the genetic variants found in different strains of inbred mice^25,30,33,37–39^. Although not inbred, HC has maintained a MutaMouse colony from a small founding population over 78 generations since 1990. Despite this, we found that there is a high level of genetic diversity present within the HC colony. In fact, the numbers of private (unshared) variants between animals sequenced from this colony ranged from 54,000 to 148,000 (Fig. 1A). Overall, our results suggest that the MutaMouse colony at HC is genetically heterogeneous and shows that new genetic variants have arisen after 78 generations. Conversely, the Covance colony had fewer private variants (24,259 to 27,277). We excluded that these results were due to the difference in coverage obtained from these two colonies (Tables 1 and S4) or to library type (Supplemental Table S5). Thus, we conclude that the reduce genetic diversity in the Covance colony is partly due to a population bottleneck experienced in the Covance colony in 2006 when the colony was restarted using a few pairs of animals.

Littermates from one inbred mouse colony reportedly vary from each other by approximately 985 SNVs genome wide^40^. We observed 50–150 fold more private SNVs between littermates. A possible explanation is that outbred colonies possess higher number of private variants when compared to inbred populations, with the important caveat that cross-study comparisons may be unreliable (e.g., different reference genomes were used, different variant calling workflows were followed). Overall, the selection of mouse strains used in an experiment requires considering the trade-offs between reducing variability in the results of toxicological assays by using inbred lines, or capturing more biological diversity to more closely reflect different responses that may exist in human populations.

Since the MutaMouse model is primarily used in genetic toxicology studies, we focused our *in silico* analysis on variants that are private to MutaMouse and carry potential phenotypic effects for genes involved in DNA repair, xenobiotic metabolism, cancer progression, and cell cycle regulation. For example, we identified a SNV within the *Ahr* gene that was present in all sequenced animals and that was predicted to affect the function of the protein. This is of special interest to research groups that regularly use aromatic hydrocarbons as model mutagens, since it may influence gene expression in response to certain chemical insults. Moreover, chemicals and drugs that are AhR agonists play important roles in carcinogenesis^41^. However, previous work indicates that the *Ahr* gene in MutaMouse has a normal phenotype because: (a) Cyp1A1, a downstream target of AhR, is highly inducible following exposure to PAHs and (b) these mice effectively metabolize aromatic compounds (a process that is regulated by AhR) resulting in very strong induction of mutations^8,42^. Additionally, there were no deleterious variants in genes involved in xenobiotic metabolism processes (e.g., cytochrome P450s). This serves as an example that *in silico* predictions, such as the biological effect of a SNV or mutation, always need phenotypic anchoring^43^. There were no potentially deleterious mutations in P53 and its key downstream targets, supporting the observation that the MutaMouse possesses an intact P53 response^44,45^. Overall, our results raise no concern about the utility of MutaMouse as a model for genetic toxicology studies.

Some mouse genomic studies have historically been limited because they used analytical methods that did not call SVs, which make up a large base-pair number component of variation between individuals^46^. The putative SVs identified in MutaMouse are far less numerous than the SNVs and short indels discovered using the HaplotypeCaller (Fig. 1B). We used multiple algorithms (CNVnator and Manta) to call distinct types of SVs. CNVnator^47^ uses a depth-based approach in which coverage is binned over a range (250 bp regions in this study) and adjacent bins are compared to identify areas of increased coverage (duplications) or decreased coverage (deletions). Manta^48^ uses split read, read pair, and read depth information to discover potential rearrangements (translocations) as well as tandem duplications, inversions, deletions, and insertions. In addition to NGS data and the associated algorithms, we also used aCGH to call CNVs (large insertions or deletions, typically on the scale >1 kb, depending on probe distances). We identified ~35,000 SVs per genome using Manta, ~4,000 CNVs using CNVnator, and ~300 CNVs using aCGH. We show these results in Fig. 2, which also shows runs of homozygosity and SNV density at a genome-wide scale for all the animals sequenced. Importantly though, we found no SVs that altered the coding sequence of toxicologically relevant genes (Table 4).

NGS and aCGH provide highly complementary data types and can be used to strengthen one another. We found that analyzing NGS coverage data together with aCGH data added precision to CNV calls generated by aCGH. We found that aCGH is highly sensitive and can accurately predict the locations of CNVs (both deletions and gains; Fig. 4A,B). However, the non-uniform spacing of array probes obscured the true nature of a subset of these events (Fig. 4C,D). Specifically, erroneous calls of single large CNVs were made based on the aCGH data, which the NGS data revealed to be composed of a complex series of small insertions and/or deletions. This is important for adding clarity to datasets; specifically, this resolves the apparently contradictory result that CNV calls from aCGH overlapped with SNV calls. Instead, these types of CNVs should be considered complex genomic regions where multiple chromosomal breaks occurred in a single span, along with potential mutations, similar to chromothripsis or other types of complex genomic rearrangements^49^. As NGS is becoming more affordable and the algorithms used for variant calling improve, both methodologies should be used to more precisely characterize SVs.

Our results are in line with a recent study^28^ that used a combination of short-read and long-read technology with the goal of improving the reference assembly of C57BL/6J (“Eve”). They were able to close previously open gaps in the assembly, resolve repeats, detect novel isoforms, and characterize structural variants with their approach. Their conclusion that genetic drift resulting from bottlenecks occurs frequently in mouse populations reflects precisely what we observed in our study. This support the concept of using different sequencing technologies in tandem to better characterize the genetics of mouse strains currently in use.

It is known that the MutaMouse λgt10-*lacZ* reporter transgene is found at a single chromosomal location on chromosome 3 (B region)^36^. Later qPCR work detailed that the transgene is present in ~29 head to tail copies^35^. The authors also identified several *in vivo* rearrangements of the transgene, although the precise breakpoints of integration, and whether the integration interrupted any genes, were not determined. We found that integration of the λgt10 transgene occurred in a complex manner and was accompanied by a large deletion of nearly 0.5 Mbp of genomic DNA from MutaMouse. The nearby long terminal repeat retrotransposon (RMER20A#LTR/ERVK) may have played a role in the integration event and was likely the reason that the genomic coordinates of the transgene were obscured when screening using short-read technology. Long-read PacBio sequence data were key to resolving the integration region (Fig. 4), since the Illumina reads adjacent to and overlapping the insertion breakpoints were too short to extend into the lambda phage shuttle vector.

Characterized of the breakpoints and integration site demonstrated that no critical genes were interrupted or deleted by transgene integration (although the lincRNA *5430434I15Rik* was deleted and predicted gene *Gm20755* was truncated). At the downstream breakpoint, there was a 2 bp microhomology that is consistent with the microhomology-mediated break-induced replication mechanism^50^. However, the upstream breakpoint consists of a 370 bp inversion in which mouse genomic sequence that originates from an area past the downstream breakpoint was placed before the insertion of four apparently random nucleotides, followed by the insertion of a transgene monomer. This is may have occurred through the hijacking of a repair process such as non-homologous end joining (NHEJ) by the foreign λgt10 DNA, although it is unclear whether the 370 bp inversion was part of an independent event. At both breakpoints, the transgene monomer is missing one of the cohesive ends, and therefore cannot be packaged into phage particles. The nonfunctional transgene monomer at the upstream breakpoint does not contain the replication origin. Our results provide some important mechanistic insight into how these random genomic integration events occur. It is becoming more apparent that structural genomic rearrangements are not uncommon in mammalian genomes and can occur via a wide range of mechanisms^51^. Our results support the hypothesis that complex structural rearrangement events require little homology to form and thus, they can form anywhere along the DNA.

Our study provides a detailed genetic analysis of the commonly used MutaMouse model that confirms its efficacy for genetic toxicology research and enhances the ability to apply new analytical options for future researchers. By sequencing multiple closely related individuals, we explored the variability in closely related mice within an outbred colony to show that a high level of diversity is present. Though we found several potentially functional SNVs, they do not appear to alter the expected functions, highlighting that such results always require phenotypic anchoring. In addition, we found that SVs did not affect coding sequences in genes for pathways relevant to genetic toxicology testing. We discovered that the genetic alteration accompanying transgene integration was a highly complex event, although it did not modify any genes. The MutaMouse genome contributes data for understanding quantitative trait loci and provide important genetic context to studies using this model.

## Methods

### Animals sequenced and experimental design

A colony of MutaMouse animals has been maintained at HC since September 1990, producing 98 generations of animals as of December 2018. Each generation is composed of the offspring from 20 to 60 females, avoiding brother and sister matings. Maintenance of this colony and the use of animals for this study were approved by the Health Canada Ottawa Animal Care Committee. All experimental procedures, including euthanasia, were conducted in accordance with relevant guidelines and regulations. We sequenced spleen DNA from five male animals from generation 78 (April 2013) to an average base depth of 63X. We used three platforms (Illumina HiSeq, Illumina NextSeq and Pacific Biosciences) and three library insert sizes (500 bp and 3 kb for Illumina, 20 kb for Pacific Biosciences). Sequencing with paired-end reads, mate pair libraries, and long-read PacBio was undertaken to increase our sensitivity for the identification of SVs.

To compare the genetic variation between geographically separated colonies of mice, we obtained two MutaMouse animals from the Covance breeding colony in the UK. The two colonies housed at Covance and HC were bred as separate lineages since the HC colony was established in 1990. However, the Covance colony experienced a founder effect when a few breeding pairs were used to restart the colony around 2006 (12 years prior to the collection of the animals used in this study). This colony was otherwise maintained in a manner similar to that of the HC colony.

### Illumina truseq and nextera mate pair library construction and sequencing

DNA was isolated from the spleens of five MutaMouse males (designated MutaMouse 1 through 5) using the QiaQuick Blood and Tissue kit (Qiagen). TruSeq paired-end libraries were prepared for all animals by shearing 1 µg of DNA with a Covaris ultrasonic disruptor followed by ligation of Illumina adapter sequences as per the manufacturer’s protocol. DNA from MutaMouse animal 2 was additionally used to prepare a Nextera Mate Pair library. Libraries were prepared by the Génome Québec Innovation Centre (Montréal, QC).

Low-coverage sequencing of MutaMouse 1 through 5 was carried out by the Génome Québec Innovation Centre (Montreal, Canada) using one lane of a HiSeq. 2500 (PE150). An entire additional HiSeq lane was used for the Nextera Mate Pair library of MutaMouse animal 2. The sequencing depth for all animals was increased by re-sequencing the same libraries in-house on a NextSeq. 500 (using the 300V2 sequencing kit) using one flow cell per animal sequenced. The two additional mice (male and female) from the Covance MutaMouse colony were used to construct Nextera Mate Pair libraries, which were then sequenced on the NextSeq. 500 as described above. All Illumina sequence reads produced were paired end, 150 bp. Data output from the sequencing are shown in Table 1.

### Pacific biosciences sequencing

DNA was isolated from MutaMouse animal 2 using phenol-chloroform extraction to obtain high molecular weight fragments for long read sequencing. This DNA was processed into a SMRTbell large insert library and sequenced using 24 SMRTcells on the PacBio RSII sequencing instrument. PacBio sequencing was carried out by Génome Québec.

### Variant calling

Variants were called using the Genome Analysis Toolkit (GATK version 3.6.0^52^ best practices). No changes to the toolkit settings were implemented except for those necessary to use the mm10 build (GRCm38) of the *Mus musculus* genome. The FASTQ files obtained from sequencing were aligned using BWA (version 0.7.12-r1039) using the maximal exact match (MEM) algorithm with duplicate marking enabled. The resulting BAM files were sorted using Samtools (version 1.3), then duplicates were removed using the Picard criteria and BAM files were merged per animal using Picard tools (version 2.5.0). GATK was used to perform base quality score recalibration and variant calling using HaplotypeCaller tool to produce gVCFs. All animals were jointly genotyped using GenotypeGVCFs, and variant quality score recalibration was performed separately for SNVs and indels using the VariantRecalibrator and ApplyRecalibration, using dbSNP for mouse v142 as the truth set of known variants. The final callset was produced by filtering variants to include 99.9% of sites in the truth set. Variants that were not marked PASS were removed from downstream analysis. Overlap between datasets was determined using BCFtools stats (version 1.3).

Runs of homozygosity (stretches of sequence for which only homozygous variant calls were made) were estimated using BCFtools roh version 1.3.

We compiled a list of 642, 1434, 408 and 45 genes involved in cancer progression, cell cycle regulation, DNA repair, and 45 xenobiotic metabolism, respectively. Cancer genes were identified from the COSMIC database, accessed Sept 11, 2016, while genes in the other pathways were identified by their gene ontology [GO] terms (Supplemental Table S2). We used this list to extract MutaMouse variants that are present in coding sequences for genes involved in toxicological functions.

### Structural variant calling

SVs including duplication or translocation breakpoints, small insertions and deletions (<50 bp), as well as large insertions and deletions (>50 bp), were identified using Manta (version 1.01; Illumina; http://github.com/Illumina/manta). This software relies on the identification of discordant paired-end reads that would normally exist in the forward-reverse (FR) orientation. Different types of structural rearrangements can be inferred by observed alignments (e.g., RR orientation indicates an inverted DNA sequence^48^). Mate pair libraries were also converted to pseudo-paired-end format by reversing the SAM flags 0 × 16 and 0 × 32 for each read, thereby converting the normal reads to the conventional FR orientation and enabling the use of Manta for mate-pair libraries. The resulting VCF files for called diploid SVs were filtered stringently using the suggested Manta filters. Furthermore, split read support for the alternate allele had to achieve a minimum sequencing depth of 20 and consist of precisely determined breakpoints.

### Copy number variant analysis using array comparative genomic hybridization and NGS

Genomic DNA was extracted from C57BL/6 spleen obtained from Jackson Laboratory (stock 000664) using the QiaQuick Blood and Tissue kit (Qiagen, Valencia, CA, USA). For each MutaMouse animal analyzed, 1 µg MutaMouse genomic DNA and 1 µg C57BL/6 reference DNA was labeled with Cy5 and Cy3, respectively (5191-3400 SureTag DNA Labelling Kit; Agilent Technologies, Cedar Creek, TX, USA). For each sample, labelled DNA was mixed and hybridized to an Agilent SurePrint G3 Mouse CGH array with 1 M probes (G4838A; Agilent Technologies) according to the manufacturer’s directions (Agilent Oligonucleotide Array-Based CGH for Genomic DNA Analysis; version 7.3). Slides were scanned using an Agilent scanner and data extracted using Agilent Feature Extraction Software v10.5. Signal intensities from Cy3 and Cy5 were then background subtracted and subjected to Lowess normalization using Rank invariant probes. Probes that were identified as outliers (i.e., affected by their position on the array) were eliminated from the dataset. CNVs were called from the probe values using R-Gada^53^.

CNVs were called from NGS data using CNVnator version 0.3.3^47^ and filtered based on the t-test (p < 0.05). A bin size of 250 bp was used for read partitioning for CNV calling. The overlap between datasets consisting of aCGH, CNVnator, and Manta calls was determined using bedtools v2.26.0. Genetic variation (SNVs and CNVs) between the five MutaMouse animals from a colony maintained at HC is reported graphically using circos plot^54^.

### Transgene breakpoint characterization, de novo assembly, and variants

We attempted to identify the location of the breakpoints using a published method^55^. However, the aligned reads were located on many different chromosomes with no obvious pattern, suggesting that transgene integration took place in a genomic region containing repetitive elements. Therefore, we used the long read lengths produced by PacBio sequencing to first align the reads to the λgt10 sequence^35^ using BWA-MEM with the -x pacbio flag enabled (equivalent to enabling the following settings: -k17 -W40 -r10 -A1 -B1 -O1 -E1 -L0). Subsequently, we aligned the subset of reads with sequence similarity to λgt10 back to the mouse genome. This resulted in the identification of reads containing regions of overlap with both mouse genomic DNA and λgt10 sequences, thereby identifying the breakpoints.

Shotgun sequence Illumina reads from MutaMouse animal 2 were aligned to the λgt10 reference sequence using BWA-MEM as described for variant calling. Aligned reads were extracted using Samtools v1.3 and assembled using SPAdes version 3.9.1. A 47,588 bp contig with 809X coverage was obtained and used for downstream analysis. Reads were re-aligned to this new reference contig using the same procedure, and variants were called using GATK HaplotypeCaller using a ploidy of 31 (i.e., 1 out of ~29 copies of the transgene in the MutaMouse should still be above the detection limit). This contig was annotated using Prokka v1.12, using default settings and the–proteins flag set to use the λ phage reference sequence NC_001416. The genes were visualized using the R package Gviz^56^.

### Comparative genomics analysis

The number of homozygous variants for each mouse from the HC and Covance colony was enumerated using BCFtools. SNPhylo^57^ was used to build a phylogenetic tree from a multi-sample VCF file. The pairwise genotype concordance for each mouse was calculated using BCFtools gtcheck. The Ensembl Variant Effect Predictor version 89.7^58^ and SnpEff version 4.1k^59^ was used to predict the consequences of variants and create annotated VCF files. For comparison to parental strains DBA/2 J and BALB/cJ, we used the strain-specific SNPs and indels found in dbSNP v142.

### Data access

The raw sequences generated for this study are available in the SRA under BioProject No. PRJNA471839.

## Supplementary information

MutaMouse_Supplemental tables
