# Supplementary material for: Whole Genome Sequencing of the Mutamouse Model Reveals Strain- and Colony-Level Variation, and Genomic Features of the Transgene Integration Site
Source: Sci Rep. 2019 Sep 24;9:13775. doi: 10.1038/s41598-019-50302-0 (PMC6760142; doi:10.1038/s41598-019-50302-0)
Supplement: Supplementary file 1 — MutaMouse_Supplemental tables [file 41598_2019_50302_MOESM1_ESM.pdf]

WHOLE GENOME SEQUENCING OF THE MUTAMOUSE MODEL REVEALS STRAIN- AND COLONY-LEVEL VARIATION, AND GENOMIC FEATURES OF THE TRANSGENE INTEGRATION SITE

Matthew J. Meier[1,2], Marc A. Beal[1], Andrew Schoenrock[1], Carole L. Yauk[1], and Francesco Marchetti[1].

[1]Environmental Health Science and Research Bureau, Health Canada, Ottawa, ON, Canada.

[2]Present address: Ecotoxicology and Wildlife Health Division, Environment and Climate Change Canada, Ottawa, ON, Canada.

MJM: matthew.meier@canada.ca

MAB: marc.beal@canada.ca

AS: Andrew.schoenrock@canada.ca

CLY: carole.yauk@canada.ca

Corresponding author:

Francesco Marchetti: francesco.marchetti@canada.ca

Environmental Health Science and Research Bureau, Tunney's Pasture, 0803A, Health Canada

Ottawa, ON, Canada, K1A 0K9

Phone: (613) 957-3137

Running title: Whole genome sequence of the MutaMouse

Supplemental Table S1. Variant statistics for MutaMouse animals from the Health Canada colony sequenced in this study.

	MutaMouse 1	MutaMouse 2	MutaMouse 3	MutaMouse 4	MutaMouse 5	MutaMouse Covance Female	MutaMouse Covance Male	Source(s) and Tools Used
total_variants	4,994,589	4,961,695	5,144,580	4,949,501	5,161,277	3,909,462	3,930,237	GATK HaplotypeCaller, bcftools stats
total SNVs	3,894,348	3,860,502	4,014,320	3,857,544	4,013,971	3,146,750	3,158,530	GATK HaplotypeCaller, bcftools stats
total deletions	533,166	531,825	546,527	527,912	557,462	368,744	373,237	GATK HaplotypeCaller, bcftools stats
total insertions	473,937	473,541	488,193	470,135	493,778	326,635	329,881	GATK HaplotypeCaller, bcftools stats
% SNVs	78%	78%	78%	78%	78%	80%	80%	GATK HaplotypeCaller, bcftools stats
other	93,138	95,827	95,540	93,910	96,066	67,333	68,589	GATK HaplotypeCaller, bcftools stats
% novel	19%	20%	19%	19%	19%	16%	16%	SnpEff
% existing	81%	80%	81%	81%	81%	84%	84%	SnpEff
private variants	81,615	72,623	119,338	54,249	148,591	24,259	27,277	SnpEff
number novel	937,004	989,828	984,316	945,768	957,461	630,584	646,769	SnpEff
all_tox_functions	234,733	223,065	251,734	234,194	256,570	186,621	187,862	Ensembl Variant Effect Predictor, COSMIC, biomaRt
all_functions deleterious	150	152	181	171	153	134	134	Ensembl Variant Effect Predictor, COSMIC, biomaRt
all_functions non_synonymous_coding	1,007	961	1,138	998	1,023	809	815	Ensembl Variant Effect Predictor, COSMIC, biomaRt
all_functions novel	37,305	36,947	40,033	37,610	40,205	24,246	24,991	Ensembl Variant Effect Predictor, COSMIC, biomaRt
all_functions novel non_synonymous_coding	76	83	98	95	83	66	75	Ensembl Variant Effect Predictor, COSMIC, biomaRt
cancer	93,708	80,922	98,330	89,172	110,565	71,057	71,513	Ensembl Variant Effect Predictor, COSMIC
cancer deleterious	52	54	67	65	56	42	43	Ensembl Variant Effect Predictor, COSMIC
cancer non_synonymous_coding	301	313	354	324	327	237	242	Ensembl Variant Effect Predictor, COSMIC
cancer novel	15,099	14,455	15,681	14,906	16,748	9,512	9,820	Ensembl Variant Effect Predictor, COSMIC
cancer novel non_synonymous_coding	34	38	45	44	39	37	44	Ensembl Variant Effect Predictor, COSMIC

cancer deleterious novel	8	10	20	20	11	20	22	Ensembl Variant Effect Predictor, COSMIC
cell_cycle	133,838	135,318	147,108	136,668	138,297	111,611	112,389	Ensembl Variant Effect Predictor, biomaRt
cell_cycle deleterious	98	96	117	102	98	91	90	Ensembl Variant Effect Predictor, biomaRt
cell_cycle non_synonymous_coding	659	598	745	621	645	541	545	Ensembl Variant Effect Predictor, biomaRt
cell_cycle novel	21,452	21,943	23,716	21,821	22,785	14,304	14,734	Ensembl Variant Effect Predictor, biomaRt
cell_cycle novel non_synonymous_coding	47	47	58	51	49	31	36	Ensembl Variant Effect Predictor, biomaRt
cell cycle novel sift deleterious	23	24	29	26	23	17	17	Ensembl Variant Effect Predictor, biomaRt
dna_repair	35,592	34,416	39,493	37,618	39,869	28,344	28,474	Ensembl Variant Effect Predictor, biomaRt
dna_repair deleterious	27	27	27	30	28	29	29	Ensembl Variant Effect Predictor, biomaRt
dna_repair non_synonymous_coding	244	222	246	238	258	198	199	Ensembl Variant Effect Predictor, biomaRt
dna_repair novel	5,567	5,475	6,055	5,802	6,156	3,663	3,748	Ensembl Variant Effect Predictor, biomaRt
dna_repair novel non_synonymous_coding	3	9	3	7	5	6	7	Ensembl Variant Effect Predictor, biomaRt
dna repair novel sift deleterious	-	4	1	3	1	3	3	Ensembl Variant Effect Predictor, biomaRt
xenobiotic_metabolism	3,483	3,295	3,469	3,326	3,278	2,932	2,934	Ensembl Variant Effect Predictor, biomaRt
xenobiotic_metabolism deleterious	2	2	2	2	2	2	2	Ensembl Variant Effect Predictor, biomaRt
xenobiotic_metabolism non_synonymous_coding	13	11	11	11	11	10	10	Ensembl Variant Effect Predictor, biomaRt
xenobiotic_metabolism novel	630	586	643	583	592	362	354	Ensembl Variant Effect Predictor, biomaRt
xenobiotic_metabolism novel non_synonymous_coding	1	1	1	1	1	1	1	Ensembl Variant Effect Predictor, biomaRt
xenobiotic novel sift deleterious	1	1	1	1	1	1	1	Ensembl Variant Effect Predictor, biomaRt
CNVnator Deletions	2,567	2,324	2,691	2,415	2,828	1,906	2,034	CNVnator
CNVnator Duplications	1,652	2,887	1,528	1,614	1,426	2,294	2,348	CNVnator
Manta Deletions	5,097	4,831	5,232	4,931	5,386	311	81	manta

**Manta Short Deletions**	11,941	10,533	12,435	11,771	12,988	705	100	manta
**Manta Translocations**	7,096	5,034	7,479	6,715	8,816	130	30	manta
**Manta Inversions**	1,052	884	1,092	1,041	1,208	28	13	manta
**Manta Insertions**	2,537	2,277	2,649	2,542	2,798	60	15	manta
**Manta Short Insertions**	4,889	3,786	5,024	4,703	5,261	140	11	manta
**Manta Tandem Duplications**	1,208	1,007	1,222	1,142	1,247	18	5	manta

Supplementary Table 2. Lists of mouse genes within pathways related to toxicology.

MGI Gene Symbol	cancer	cell cycle	DNA repair	xenobiotic metabolism
1500015O10Rik		cell cycle		
1700123I01Rik		cell cycle		
1810011O10Rik		cell cycle		
2610002M06Rik		cell cycle		
2810417H13Rik		cell cycle	DNA repair	
4930544G11Rik	cancer			
4933406J08Rik		cell cycle		
4933427D14Rik		cell cycle		
Aatf		cell cycle		
Abcb1a		cell cycle		
Abi1	cancer			
Abl1	cancer	cell cycle	DNA repair	
Abl2	cancer			
Ackr3	cancer			
Acsl1				xenobiotic metabolism
Acsl3	cancer			
Acsl6	cancer			
Actl6a			DNA repair	
Actr2		cell cycle		
Actr3		cell cycle		
Actr5			DNA repair	
Actr8		cell cycle	DNA repair	
Acvr1	cancer	cell cycle		
Acvr1b		cell cycle		
Adam17		cell cycle		
Adarb1		cell cycle		
Adcyap1		cell cycle		
Afap1l2		cell cycle		
Aff1	cancer			
Aff3	cancer			
Aff4	cancer			
Ago4		cell cycle		
Ahctf1		cell cycle		
Ahr		cell cycle		xenobiotic metabolism
Ahrr				xenobiotic metabolism
Aif1		cell cycle		
Aip				xenobiotic metabolism
Ajuba		cell cycle		
Ak1		cell cycle		
Akap8		cell cycle		
Akap8l		cell cycle		
Akap9	cancer			
Akr1c12				xenobiotic metabolism
Akr1c13				xenobiotic metabolism
Akt1	cancer	cell cycle		
Akt2	cancer			

Aldh2	cancer		
Alk	cancer		
Alkbh1			DNA repair
Alkbh2			DNA repair
Alkbh3			DNA repair
Alkbh4		cell cycle	
Alox8		cell cycle	
Amer1	cancer		
Anapc1		cell cycle	
Anapc10		cell cycle	
Anapc11		cell cycle	
Anapc13		cell cycle	
Anapc15		cell cycle	
Anapc16		cell cycle	
Anapc2		cell cycle	
Anapc4		cell cycle	
Anapc5		cell cycle	
Anapc7		cell cycle	
Angel2		cell cycle	
Ank3		cell cycle	
Ankle2		cell cycle	
Ankrd17		cell cycle	
Ankrd53		cell cycle	
Anln		cell cycle	
Anp32b		cell cycle	
Anxa1		cell cycle	
Anxa11		cell cycle	
Ap5s1			DNA repair
Ap5z1			DNA repair
Apbb1		cell cycle	DNA repair
Apbb2		cell cycle	
Apc	cancer	cell cycle	
Apex1		cell cycle	DNA repair
Apex2		cell cycle	DNA repair
Apitd1		cell cycle	DNA repair
Aplf			DNA repair
App		cell cycle	
Appl1		cell cycle	
Appl2		cell cycle	
Aptx			DNA repair
Ar	cancer		
Arf6		cell cycle	
Arhgap26	cancer		
Arhgef10		cell cycle	
Arhgef12	cancer		
Arhgef2		cell cycle	
Arid1a	cancer		
Arid1b	cancer		

Arid2	cancer			
Arl2		cell cycle		
Arl3		cell cycle		
Arl8a		cell cycle		
Arl8b		cell cycle		
Arnt	cancer			
Arntl		cell cycle		
Arpp19		cell cycle		
As3mt				xenobiotic metabolism
Ascc3			DNA repair	
Ascl1		cell cycle		
Asf1a			DNA repair	
Asns		cell cycle		
Aspm		cell cycle		
Aspscr1	cancer			
Asun		cell cycle		
Asxl1	cancer			
Asz1		cell cycle		
Atad5		cell cycle		
Atf1	cancer			
Atf2		cell cycle		
Atf5		cell cycle		
Atic	cancer			
Atm	cancer	cell cycle	DNA repair	
Atp1a1	cancer			
Atp2b3	cancer			
Atp2b4		cell cycle		
Atr	cancer	cell cycle	DNA repair	
Atraid		cell cycle		
Atrx	cancer	cell cycle	DNA repair	
Aunip		cell cycle		
Aurka		cell cycle		
Aurkb		cell cycle		
Aurkc		cell cycle		
Avpi1		cell cycle		
Axin1	cancer			
Axin2	cancer	cell cycle	DNA repair	
AY074887		cell cycle		
Azi2		cell cycle		
B2m	cancer			
Babam1		cell cycle	DNA repair	
Bach1		cell cycle	DNA repair	
Bad		cell cycle		
Bag6		cell cycle		
Bak1		cell cycle		
Banp		cell cycle		
Bap1	cancer	cell cycle		
Bard1			DNA repair	

Bax		cell cycle	
Baz1b			DNA repair
Bbs4		cell cycle	
BC004004		cell cycle	
BC029214			DNA repair
Bcat1		cell cycle	
Bccip		cell cycle	DNA repair
Bcl10	cancer		
Bcl11a	cancer		
Bcl11b	cancer		
Bcl2	cancer	cell cycle	
Bcl2l1		cell cycle	
Bcl2l11		cell cycle	
Bcl3	cancer		
Bcl6	cancer		
Bcl7a	cancer		
Bcl9	cancer		
Bcor	cancer		
Bcorl1	cancer		
Bcr	cancer	cell cycle	
Becn1		cell cycle	
Bex2		cell cycle	
Bid		cell cycle	
Bin1		cell cycle	
Bin3		cell cycle	
Birc2		cell cycle	
Birc3	cancer		
Birc5		cell cycle	
Birc6		cell cycle	
Birc7		cell cycle	
Bivm			DNA repair
Blcap		cell cycle	
Blm	cancer	cell cycle	DNA repair
Bmp2		cell cycle	
Bmp4		cell cycle	
Bmp7		cell cycle	
Bmpr1a	cancer		
Bod1		cell cycle	
Bod1l			DNA repair
Boll		cell cycle	
Bop1		cell cycle	
Bora		cell cycle	
Braf	cancer		
Brca1	cancer	cell cycle	DNA repair
Brca2	cancer	cell cycle	DNA repair
Brcc3		cell cycle	DNA repair
Brd3	cancer		
Brd4	cancer	cell cycle	

Brd7		cell cycle	
Brdt		cell cycle	
Bre		cell cycle	DNA repair
Brinp1		cell cycle	
Brinp2		cell cycle	
Brinp3		cell cycle	
Brip1	cancer	cell cycle	DNA repair
Brsk1		cell cycle	
Brsk2		cell cycle	
Btc		cell cycle	
Btg1	cancer		
Btg3		cell cycle	
Btg4		cell cycle	
Btn2a2		cell cycle	
Btrc		cell cycle	
Bub1		cell cycle	
Bub1b	cancer	cell cycle	
Bub3		cell cycle	
C2cd3		cell cycle	
Cables1		cell cycle	
Cables2		cell cycle	
Cacna1d	cancer		
Cacul1		cell cycle	
Calm1		cell cycle	
Calm2		cell cycle	
Calm3		cell cycle	
Calr	cancer	cell cycle	
Camk1		cell cycle	
Camk2a		cell cycle	
Camk2b		cell cycle	
Camk2d		cell cycle	
Camk2g		cell cycle	
Camta1	cancer		
Cant1	cancer		
Capn3		cell cycle	
Card11	cancer		
Cars	cancer		
Casc5	cancer	cell cycle	
Casp2		cell cycle	
Casp3		cell cycle	
Casp8	cancer		
Casp8ap2		cell cycle	
Cast		cell cycle	
Cbfa2t3	cancer		
Cbfb	cancer		
Cbl	cancer		
Cblb	cancer		
Cblc	cancer		

Cbx8			DNA repair
Ccar1		cell cycle	
Ccar2		cell cycle	
Ccdc124		cell cycle	
Ccdc155		cell cycle	DNA repair
Ccdc6	cancer		
Ccdc67		cell cycle	
Ccdc79		cell cycle	
Ccdc8		cell cycle	
Ccna1		cell cycle	
Ccna2		cell cycle	
Ccnb1		cell cycle	
Ccnb1ip1	cancer	cell cycle	
Ccnb2		cell cycle	
Ccnb3		cell cycle	
Ccnc		cell cycle	
Ccnd1	cancer	cell cycle	
Ccnd2	cancer	cell cycle	
Ccnd3	cancer	cell cycle	
Ccndbp1		cell cycle	
Ccne1	cancer	cell cycle	
Ccne2		cell cycle	
Ccnf		cell cycle	
Ccng1		cell cycle	
Ccng2		cell cycle	
Ccnh		cell cycle	
Ccni		cell cycle	
Ccnk		cell cycle	
Ccnl1		cell cycle	
Ccnl2		cell cycle	
Ccno		cell cycle	DNA repair
Ccnt1		cell cycle	
Ccnt2		cell cycle	
Ccny		cell cycle	
Ccp110		cell cycle	
Ccpg1		cell cycle	
Ccpg1os	cancer		
Ccsap		cell cycle	
Cd274	cancer		
Cd28		cell cycle	
Cd2ap		cell cycle	
Cd74	cancer		
Cd79a	cancer		
Cd79b	cancer		
Cdc123		cell cycle	
Cdc14a		cell cycle	
Cdc14b		cell cycle	DNA repair
Cdc16		cell cycle	

Cdc20		cell cycle	
Cdc23		cell cycle	
Cdc25a		cell cycle	
Cdc25b		cell cycle	
Cdc25c		cell cycle	
Cdc26		cell cycle	
Cdc27		cell cycle	
Cdc34		cell cycle	
Cdc42		cell cycle	
Cdc45		cell cycle	DNA repair
Cdc5l		cell cycle	DNA repair
Cdc6		cell cycle	
Cdc7		cell cycle	DNA repair
Cdc73	cancer	cell cycle	
Cdca2		cell cycle	
Cdca3		cell cycle	
Cdca5		cell cycle	DNA repair
Cdca8		cell cycle	
Cdh1	cancer		
Cdh11	cancer		
Cdk1		cell cycle	
Cdk10		cell cycle	
Cdk11b		cell cycle	
Cdk12	cancer		
Cdk14		cell cycle	
Cdk2		cell cycle	DNA repair
Cdk20		cell cycle	
Cdk4	cancer	cell cycle	
Cdk5		cell cycle	
Cdk5r1		cell cycle	
Cdk5rap1		cell cycle	
Cdk5rap2		cell cycle	
Cdk5rap3		cell cycle	
Cdk6	cancer	cell cycle	
Cdk7		cell cycle	DNA repair
Cdk9		cell cycle	DNA repair
Cdkn1a		cell cycle	
Cdkn1b	cancer	cell cycle	
Cdkn1c		cell cycle	
Cdkn2a		cell cycle	
Cdkn2b		cell cycle	
Cdkn2c	cancer	cell cycle	
Cdkn2d		cell cycle	DNA repair
Cdkn3		cell cycle	
Cdt1		cell cycle	
Cdx2	cancer		
Cebpa	cancer	cell cycle	
Cebpg			DNA repair

Cenpa		cell cycle	
Cenpc1		cell cycle	
Cenpe		cell cycle	
Cenpf		cell cycle	
Cenph		cell cycle	
Cenpj		cell cycle	
Cenpn		cell cycle	
Cenpt		cell cycle	
Cenpv		cell cycle	
Cenpw		cell cycle	
Cep120		cell cycle	
Cep126		cell cycle	
Cep131		cell cycle	
Cep135		cell cycle	
Cep152		cell cycle	
Cep164		cell cycle	DNA repair
Cep192		cell cycle	
Cep250		cell cycle	
Cep55		cell cycle	
Cep57		cell cycle	
Cep57l1		cell cycle	
Cep63		cell cycle	
Cep72		cell cycle	
Cep76		cell cycle	
Cep85		cell cycle	
Cep89	cancer		
Cep97		cell cycle	
Cetn1		cell cycle	
Cetn2		cell cycle	DNA repair
Cetn3		cell cycle	
Cfl1		cell cycle	
Cgref1		cell cycle	
Cgrrf1		cell cycle	
Chaf1a		cell cycle	DNA repair
Chaf1b		cell cycle	DNA repair
Champ1		cell cycle	
Chchd4			DNA repair
Chchd7	cancer		
Chd1l			DNA repair
Chd3		cell cycle	
Chd4	cancer	cell cycle	
Chek1		cell cycle	DNA repair
Chek2	cancer	cell cycle	DNA repair
Chfr		cell cycle	
Chic2	cancer		
Chmp1a		cell cycle	
Chmp1b		cell cycle	
Chmp2a		cell cycle	

Chmp2b		cell cycle	
Chmp3		cell cycle	
Chmp4b		cell cycle	
Chmp4c		cell cycle	
Chmp5		cell cycle	
Chmp6		cell cycle	
Chmp7		cell cycle	
Chordc1		cell cycle	
Chrna4			DNA repair
Chtf18		cell cycle	
Chtf8		cell cycle	
Cib1		cell cycle	
Cic	cancer		
Ciita	cancer		
Cinp		cell cycle	DNA repair
Cirbp		cell cycle	
Cit		cell cycle	
Cited2		cell cycle	
Ckap2		cell cycle	
Ckap5		cell cycle	
Cks1b		cell cycle	
Cks1brt		cell cycle	
Cks2		cell cycle	
Clasp1		cell cycle	
Clasp2		cell cycle	
Clgn		cell cycle	
Clip1	cancer		
Clock		cell cycle	
Clp1	cancer		
Clspn		cell cycle	DNA repair
Cltc	cancer	cell cycle	
Cnbp	cancer		
Cnot3	cancer		
Cnppd1		cell cycle	
Cntd1		cell cycle	
Cntrl	cancer	cell cycle	
Cntrob		cell cycle	
Col1a1	cancer		
Col2a1	cancer		
Cops5		cell cycle	
Cox6c	cancer		
Cpeb1		cell cycle	
Cradd		cell cycle	
Creb1	cancer		
Creb3l1	cancer		
Creb3l2	cancer		
Crebbp	cancer	cell cycle	
Crlf2	cancer		

Crlf3		cell cycle		
Crocc		cell cycle		
Crtc1	cancer			
Crtc3	cancer			
Cry1		cell cycle		
Cryz				xenobiotic metabolism
Csf3r	cancer			
Csnk1a1		cell cycle		
Csnk1d		cell cycle		
Csnk2a1		cell cycle		
Csnk2a2		cell cycle		
Cspp1		cell cycle		
Csrp2bp		cell cycle		
Ctbp1		cell cycle		
Ctc1		cell cycle		
Ctcf	cancer			
Ctdp1		cell cycle		
Ctdsp1		cell cycle		
Ctdsp2		cell cycle		
Ctdspl		cell cycle		
Ctgf		cell cycle		
Ctnnb1	cancer	cell cycle		
Cts7		cell cycle		
Cul3		cell cycle		
Cul4a		cell cycle	DNA repair	
Cul4b		cell cycle	DNA repair	
Cul7		cell cycle		
Cul9		cell cycle		
Cux1	cancer			
Cuzd1		cell cycle		
Cxcr4	cancer			
Cxcr5		cell cycle		
Cyld	cancer	cell cycle		
Cyp1a1		cell cycle		xenobiotic metabolism
Cyp1b1				xenobiotic metabolism
Cyp26a1				xenobiotic metabolism
Cyp26b1		cell cycle		xenobiotic metabolism
Cyp27b1		cell cycle		
Cyp2b10				xenobiotic metabolism
Cyp2e1				xenobiotic metabolism
Cyp2f2				xenobiotic metabolism
Cyp2w1				xenobiotic metabolism
D1Pas1	cancer			
D2Wsu81e		cell cycle		
D630045J12Rik	cancer			
D7Ertd443e		cell cycle		
Dab2ip		cell cycle		
Dach1		cell cycle		

Dact1		cell cycle	
Dapk3		cell cycle	
Daxx	cancer	cell cycle	
Dazl		cell cycle	
Dbf4		cell cycle	
Dclre1a		cell cycle	DNA repair
Dclre1b		cell cycle	DNA repair
Dclre1c			DNA repair
Dctn1	cancer		
Dctn2		cell cycle	
Dctn3		cell cycle	
Dctn6		cell cycle	
Ddb1		cell cycle	DNA repair
Ddb2	cancer		DNA repair
Ddias		cell cycle	
Ddit3	cancer	cell cycle	
Ddr2	cancer		
Ddx1			DNA repair
Ddx10	cancer		
Ddx11		cell cycle	
Ddx39b		cell cycle	
Ddx3x	cancer	cell cycle	
Ddx4		cell cycle	
Ddx5	cancer		
Ddx6	cancer		
Dek	cancer		DNA repair
Dicer1	cancer	cell cycle	
Dis3l2		cell cycle	
Dixdc1		cell cycle	
Dlg1		cell cycle	
Dlgap5		cell cycle	
Dmap1			DNA repair
Dmc1		cell cycle	DNA repair
Dmrt1		cell cycle	
Dmrtc2		cell cycle	
Dmtf1		cell cycle	
Dna2		cell cycle	DNA repair
Dnajb1	cancer		
Dnm2	cancer		
Dnmt3a	cancer		
Donson		cell cycle	
Dot1l		cell cycle	DNA repair
Dpep3		cell cycle	
Drd2		cell cycle	
Drd3		cell cycle	
Drosha	cancer		
Dscc1		cell cycle	
Dsn1		cell cycle	

Dstn		cell cycle	
Dtl		cell cycle	DNA repair
Dtx3l			DNA repair
Dusp1		cell cycle	
Dusp3		cell cycle	
Dync1h1		cell cycle	
Dync1li1		cell cycle	
Dynlt1b		cell cycle	
Dynlt3		cell cycle	
E2f1		cell cycle	
E2f2		cell cycle	
E2f3		cell cycle	
E2f4		cell cycle	
E2f5		cell cycle	
E2f6		cell cycle	
E2f7		cell cycle	
E2f8		cell cycle	
E430025E21Rik		cell cycle	
E4f1		cell cycle	
Ebf1	cancer		
Ecd		cell cycle	
Ect2		cell cycle	
Ect2l	cancer		
Edn1		cell cycle	
Edn3		cell cycle	
Eef1e1			DNA repair
Eepd1			DNA repair
Egf		cell cycle	
Egfr	cancer	cell cycle	DNA repair
Ehmt2		cell cycle	
Eid1		cell cycle	
Eid3			DNA repair
Eif2ak4		cell cycle	
Eif3e	cancer		
Eif4a2	cancer		
Eif4e		cell cycle	
Eif4ebp1		cell cycle	
Eif4g3		cell cycle	
Elf4	cancer		
Elk4	cancer		
Ell	cancer		
Eln	cancer		
Eme1		cell cycle	DNA repair
Eme2		cell cycle	DNA repair
Eml1		cell cycle	
Eml4	cancer		
Emsy			DNA repair
Endov			DNA repair

Ensa		cell cycle	
Ep300	cancer	cell cycle	
Epc2			DNA repair
Epgn		cell cycle	
Eps15	cancer		
Eps8		cell cycle	
Erbb2	cancer		
Erbb3	cancer		
Erbb4	cancer		
Erc1	cancer		
Ercc1		cell cycle	DNA repair
Ercc2	cancer	cell cycle	DNA repair
Ercc3	cancer	cell cycle	DNA repair
Ercc4	cancer	cell cycle	DNA repair
Ercc5	cancer		DNA repair
Ercc6			DNA repair
Ercc6l		cell cycle	
Ercc6l2			DNA repair
Ercc8			DNA repair
Ereg		cell cycle	
Erg	cancer		
Erh		cell cycle	
Ern1		cell cycle	
Ern2		cell cycle	
Esco1		cell cycle	
Esco2		cell cycle	DNA repair
Espl1		cell cycle	
Esr1	cancer	cell cycle	
Etnk1	cancer		
Etv1	cancer		
Etv4	cancer		
Etv5	cancer		
Etv6	cancer		
Evi5		cell cycle	
Ewsr1	cancer		
Exd1		cell cycle	
Exd2			DNA repair
Exo1		cell cycle	DNA repair
Exo5			DNA repair
Ext1	cancer		
Ext2	cancer		
Eya1		cell cycle	DNA repair
Eya2			DNA repair
Eya3			DNA repair
Eya4			DNA repair
Ezh2	cancer	cell cycle	
Ezr	cancer	cell cycle	
Faap100			DNA repair

Faap20			DNA repair
Faap24			DNA repair
Fam131b	cancer		
Fam175a		cell cycle	DNA repair
Fam175b		cell cycle	
Fam178a		cell cycle	DNA repair
Fam20b		cell cycle	
Fam32a		cell cycle	
Fam46c	cancer		
Fam58b		cell cycle	
Fam64a		cell cycle	
Fam83d		cell cycle	
Fan1			DNA repair
Fanca	cancer	cell cycle	DNA repair
Fancb			DNA repair
Fancc	cancer		DNA repair
Fancd2	cancer	cell cycle	DNA repair
Fance	cancer		
Fancf	cancer		DNA repair
Fancg	cancer		DNA repair
Fanci		cell cycle	DNA repair
Fancl			DNA repair
Fancm		cell cycle	DNA repair
Fap		cell cycle	
Fas	cancer		
Fat1	cancer		
Fat4	cancer		
Fbxl15		cell cycle	
Fbxl7		cell cycle	
Fbxo11	cancer		
Fbxo18			DNA repair
Fbxo31		cell cycle	
Fbxo4		cell cycle	
Fbxo43		cell cycle	
Fbxo5		cell cycle	
Fbxo6			DNA repair
Fbxo7		cell cycle	
Fbxw11		cell cycle	
Fbxw5		cell cycle	
Fbxw7	cancer	cell cycle	
Fcgr2b	cancer		
Fcgr3	cancer		
Fem1b		cell cycle	
Fen1			DNA repair
Fer		cell cycle	
Fev	cancer		
Fgf10		cell cycle	DNA repair
Fgf2		cell cycle	

Fgf8		cell cycle		
Fgfr1	cancer	cell cycle		
Fgfr1op	cancer			
Fgfr2	cancer	cell cycle		
Fgfr3	cancer	cell cycle		
Fgfr4	cancer			
Fggy		cell cycle		
Fh1	cancer			
Fhit	cancer			
Fhl1		cell cycle		
Fign		cell cycle	DNA repair	
Fignl1		cell cycle	DNA repair	
Fignl2			DNA repair	
Fip1l1	cancer			
Fkbp6		cell cycle		
Flcn	cancer	cell cycle		
Fli1	cancer			
Flna		cell cycle		
Flt3	cancer			
Flt3l		cell cycle		
Flt4	cancer			
Fmn2		cell cycle		
Fmo2				xenobiotic metabolism
Fn3k		cell cycle		
Fn3krp		cell cycle		
Fnbp1	cancer			
Fnta		cell cycle		
Fntb		cell cycle		
Fosl1		cell cycle		
Foxa1	cancer	cell cycle		
Foxc1		cell cycle		
Foxe3		cell cycle		
Foxg1		cell cycle		
Foxl2	cancer			
Foxm1		cell cycle	DNA repair	
Foxn3		cell cycle		
Foxo1	cancer			
Foxo3	cancer			
Foxo4	cancer	cell cycle		
Foxp1	cancer			
Fsd1		cell cycle		
Fstl3	cancer			
Fto			DNA repair	
Fubp1	cancer			
Fus	cancer			
Fzd3		cell cycle		
Fzr1		cell cycle	DNA repair	
Gadd45a		cell cycle		

Gadd45b		cell cycle	
Gadd45g		cell cycle	
Gadd45gip1		cell cycle	
Gak		cell cycle	
Gas1		cell cycle	
Gas2		cell cycle	
Gas2l1		cell cycle	
Gas7	cancer		
Gata1	cancer		
Gata2	cancer		
Gata3	cancer	cell cycle	
Gata6		cell cycle	
Gbf1		cell cycle	
Gck		cell cycle	
Gdpd5		cell cycle	
Gem		cell cycle	
Gen1		cell cycle	DNA repair
Ggn			DNA repair
Gigyf2		cell cycle	
Gins1		cell cycle	
Gins2		cell cycle	DNA repair
Gins4			DNA repair
Gipc1		cell cycle	
Git1		cell cycle	
Gm10696	cancer		
Gm10697	cancer		
Gm14459	cancer		
Gm20431			DNA repair
Gm21876	cancer		
Gm37596	cancer		
Gm4778	cancer		
Gm4858	cancer		
Gm5286	cancer		
Gm5611	cancer		
Gm5751	cancer		
Gm5773	cancer		
Gm6592	cancer		
Gm6768	cancer		
Gm9117	cancer		
Gm9125	cancer		
Gm960		cell cycle	
Gmnc		cell cycle	
Gmnn		cell cycle	
Gmps	cancer		
Gna11	cancer		
Gnai1		cell cycle	
Gnai2		cell cycle	
Gnai3		cell cycle	

Gnaq	cancer			
Gnas	cancer			
Golga2		cell cycle		
Golga5	cancer			
Gopc	cancer			
Gpc3	cancer			
Gper1		cell cycle		
Gphn	cancer			
Gpr132		cell cycle		
Gpr3		cell cycle		
Gpsm2		cell cycle		
**Grand Total**	**641**	**1432**	**407**	**45**
Grin1				xenobiotic metabolism
Grin2a	cancer			
Grk5		cell cycle		
Gsg2		cell cycle		
Gsk3b		cell cycle		
Gspt2		cell cycle		
Gstm2				xenobiotic metabolism
Gstm4				xenobiotic metabolism
Gstm5				xenobiotic metabolism
Gstm7				xenobiotic metabolism
Gsto1				xenobiotic metabolism
Gsto2				xenobiotic metabolism
Gstt1				xenobiotic metabolism
Gtf2h1			DNA repair	
Gtf2h2			DNA repair	
Gtf2h3			DNA repair	
Gtf2h4			DNA repair	
Gtf2h5			DNA repair	
Gtpbp4		cell cycle		
H1foo		cell cycle		
H2afx		cell cycle	DNA repair	
H2afy		cell cycle		
H2-Bl	cancer			
H3f3b	cancer			
Hacd1		cell cycle		
Hace1		cell cycle		
Haus1		cell cycle		
Haus2		cell cycle		
Haus3		cell cycle		
Haus4		cell cycle		
Haus5		cell cycle		
Haus6		cell cycle		
Haus7		cell cycle		
Haus8		cell cycle		
Hcfc1		cell cycle		
Hdac3		cell cycle		

Hdac8		cell cycle		
Heca		cell cycle		
Helb			DNA repair	
Hells		cell cycle		
Helq			DNA repair	
Hepacam		cell cycle		
Hepacam2		cell cycle		
Herc2			DNA repair	
Herpud1	cancer			
Hes1		cell cycle		
Hexim1		cell cycle		
Hexim2		cell cycle		
Hey1	cancer			
Hfm1		cell cycle		
Hhex		cell cycle		
Hif1a	cancer			
Hinfp		cell cycle	DNA repair	
Hip1	cancer			
Hist1h3i	cancer			
Hist3h2a			DNA repair	
Hjurp		cell cycle		
Hk1		cell cycle		
Hk2		cell cycle		
Hk3		cell cycle		
Hlf	cancer			
Hltf			DNA repair	
Hmg20b		cell cycle		
Hmga1	cancer		DNA repair	
Hmga1-rs1	cancer			
Hmga2	cancer	cell cycle	DNA repair	
Hmgb1		cell cycle	DNA repair	
Hmgn1			DNA repair	
Hnf1a	cancer			
Hnf4a		cell cycle		xenobiotic metabolism
Hnrnpa2b1	cancer			
Hook3	cancer			
Hormad1		cell cycle		
Hormad2		cell cycle		
Hoxa11	cancer			
Hoxa13	cancer	cell cycle		
Hoxa9	cancer			
Hoxc11	cancer			
Hoxc13	cancer			
Hoxd11	cancer			
Hoxd13	cancer			
Hpgd		cell cycle		
Hras	cancer	cell cycle		
Hsf1		cell cycle		

Hsp90aa1	cancer		
Hsp90ab1	cancer		
Hspa1a			DNA repair
Hspa2		cell cycle	
Hspa8		cell cycle	
Htatip2		cell cycle	
Htt		cell cycle	
Hus1		cell cycle	DNA repair
Hus1b		cell cycle	DNA repair
Huwe1			DNA repair
Id2		cell cycle	
Id3		cell cycle	
Id4		cell cycle	
Idh1	cancer		
Idh2	cancer		
Ier3		cell cycle	DNA repair
Ifng		cell cycle	
Ifnz		cell cycle	
Igf1		cell cycle	
Igf1r		cell cycle	
Igf2		cell cycle	
Ikbkb	cancer		
Ikzf1	cancer	cell cycle	
Il12a		cell cycle	
Il12b		cell cycle	
Il1a		cell cycle	
Il1b		cell cycle	
Il2	cancer		
Il21r	cancer		
Il6st	cancer		
Il7r	cancer		
Ilk		cell cycle	
Ilkap		cell cycle	
Inca1		cell cycle	
Incenp		cell cycle	
Ing1		cell cycle	
Ing2		cell cycle	
Ing4		cell cycle	
Inha		cell cycle	
Inhba		cell cycle	
Inip			DNA repair
Ino80		cell cycle	DNA repair
Ino80b			DNA repair
Ino80c			DNA repair
Ino80d			DNA repair
Ins2		cell cycle	
Insc		cell cycle	
Insm1		cell cycle	

Insr		cell cycle	
Ints3		cell cycle	DNA repair
Ints7		cell cycle	
Iqgap3		cell cycle	
Irf1		cell cycle	
Irf4	cancer		
Irf6		cell cycle	
Ist1		cell cycle	
Itgb1		cell cycle	
Itgb3bp		cell cycle	
Itk	cancer		
Jade1		cell cycle	
Jak1	cancer		
Jak2	cancer		
Jak3	cancer		
Jazf1	cancer		
Jmy		cell cycle	DNA repair
Jtb		cell cycle	
Jun	cancer	cell cycle	
Junb		cell cycle	
Jund		cell cycle	
Kank2		cell cycle	
Kat2b		cell cycle	
Kat5			DNA repair
Kat6a	cancer		
Kat6b	cancer		
Katna1		cell cycle	
Katnb1		cell cycle	
Kcna5		cell cycle	
Kcnh5		cell cycle	
Kcnj5	cancer		
Kctd11		cell cycle	
Kdm1a			DNA repair
Kdm2a			DNA repair
Kdm4d			DNA repair
Kdm5a	cancer		
Kdm5c	cancer		
Kdm6a	cancer		
Kdm8		cell cycle	
Kdr	cancer		
Kdsr	cancer		
Keap1	cancer		
Khdc3		cell cycle	
Khdrbs1		cell cycle	
Kif11		cell cycle	
Kif13a		cell cycle	
Kif14		cell cycle	
Kif18a		cell cycle	

Kif18b		cell cycle	
Kif20a		cell cycle	
Kif20b		cell cycle	
Kif22		cell cycle	DNA repair
Kif23		cell cycle	
Kif2a		cell cycle	
Kif2b		cell cycle	
Kif2c		cell cycle	
Kif3b		cell cycle	
Kif4		cell cycle	
Kif5b	cancer		
Kifc1		cell cycle	
Kin			DNA repair
Kit	cancer		
Kiz		cell cycle	
Klf11		cell cycle	
Klf4	cancer		
Klf6	cancer		
Klhdc3		cell cycle	
Klhl13		cell cycle	
Klhl21		cell cycle	
Klhl22		cell cycle	
Klhl42		cell cycle	
Klhl9		cell cycle	
Klk1	cancer		
Klk1b1	cancer		
Klk1b11	cancer		
Klk1b16	cancer		
Klk1b21	cancer		
Klk1b22	cancer		
Klk1b24	cancer		
Klk1b26	cancer		
Klk1b27	cancer		
Klk1b3	cancer		
Klk1b4	cancer		
Klk1b5	cancer		
Klk1b8	cancer		
Klk1b9	cancer		
Kmt2a	cancer		
Kmt2c	cancer		
Kmt2d	cancer		
Kmt2e		cell cycle	
Kmt5a		cell cycle	
Knstrn		cell cycle	
Kntc1		cell cycle	
Kpnb1		cell cycle	
Kras	cancer		
Krtap21-1		cell cycle	

Ktn1	cancer			
L3mbtl1		cell cycle		
Larp7		cell cycle		
Lasp1	cancer			
Lats1		cell cycle		
Lats2		cell cycle		
Lck	cancer			
Lcmt1		cell cycle		
Lcp1	cancer			
Lef1	cancer			
Lemd3		cell cycle		
Lep		cell cycle		
Lfng		cell cycle		
Lhfp	cancer			
Lif		cell cycle		
Lifr	cancer			
Lig1		cell cycle	DNA repair	
Lig3		cell cycle	DNA repair	
Lig4		cell cycle	DNA repair	
Lin37		cell cycle		
Lin54		cell cycle		
Lin9		cell cycle		
Llgl1		cell cycle		
Llgl2		cell cycle		
Lmln		cell cycle		
Lmna	cancer			
Lmnb1		cell cycle		
Lmo1	cancer			
Lmo2	cancer			
Lpo				xenobiotic metabolism
Lpp	cancer			
Lrig3	cancer			
Lrp1b	cancer			
Lrp5		cell cycle		
Lrp6		cell cycle		
Lrrcc1		cell cycle		
Lsm10		cell cycle		
Lsm11		cell cycle		
Lsm14a	cancer			
Lyl1	cancer			
Lztr1	cancer			
Lzts1		cell cycle		
Lzts2		cell cycle		
M1ap		cell cycle		
Mad1l1		cell cycle		
Mad2l1		cell cycle		
Mad2l1bp		cell cycle		
Mad2l2		cell cycle	DNA repair	

Madd		cell cycle	
Maea		cell cycle	
Mael		cell cycle	
Maf	cancer		
Mafb	cancer		
Magi2		cell cycle	
Malt1	cancer		
Maml2	cancer		
Map10		cell cycle	
Map2k1	cancer	cell cycle	
Map2k2	cancer		
Map2k4	cancer		
Map3k1	cancer		
Map3k13	cancer		
Map3k8		cell cycle	
Map4		cell cycle	
Map9		cell cycle	
Mapk1	cancer	cell cycle	
Mapk12		cell cycle	
Mapk13		cell cycle	
Mapk14		cell cycle	
Mapk1ip1		cell cycle	
Mapk3		cell cycle	
Mapk4		cell cycle	
Mapk6		cell cycle	
Mapk7		cell cycle	
Mapkapk2		cell cycle	
Mapre1		cell cycle	
Mapre2		cell cycle	
Mapre3		cell cycle	
Marf1		cell cycle	DNA repair
Mark4		cell cycle	
Marveld1		cell cycle	
Mastl		cell cycle	
Mau2		cell cycle	
Max	cancer		
Mbd4		cell cycle	DNA repair
Mc1r			DNA repair
Mcidas		cell cycle	
Mcm2		cell cycle	
Mcm3		cell cycle	
Mcm4		cell cycle	
Mcm5		cell cycle	
Mcm6		cell cycle	
Mcm7		cell cycle	
Mcm8		cell cycle	DNA repair
Mcm9			DNA repair
Mcmbp		cell cycle	

Mcph1		cell cycle	
Mcrs1			DNA repair
Mcts1		cell cycle	
Mdc1		cell cycle	DNA repair
Mdm1		cell cycle	
Mdm2	cancer	cell cycle	
Mdm4	cancer	cell cycle	
Mecom	cancer	cell cycle	
Med1		cell cycle	
Med12	cancer		
Med25		cell cycle	
Mei1		cell cycle	
Mei4		cell cycle	
Meig1		cell cycle	
Meikin		cell cycle	
Meiob		cell cycle	DNA repair
Meioc		cell cycle	DNA repair
Melk		cell cycle	
Men1	cancer	cell cycle	
Mepce		cell cycle	
Met	cancer	cell cycle	
Mgme1			DNA repair
Mgmt			DNA repair
Mif		cell cycle	
Miip		cell cycle	
Mir124a-1		cell cycle	
Mir124a-2		cell cycle	
Mir124a-3		cell cycle	
Mir16-1		cell cycle	
Mir214		cell cycle	
Mir26a-1		cell cycle	
Mir26a-2		cell cycle	
Mir26b		cell cycle	
Mir744		cell cycle	
Mis12		cell cycle	
Mis18a		cell cycle	
Mis18bp1		cell cycle	
Misp		cell cycle	
Mitd1		cell cycle	
Mitf	cancer		
Mki67		cell cycle	
Mkl1	cancer		
Mlf1	cancer	cell cycle	
Mlh1	cancer	cell cycle	DNA repair
Mlh3		cell cycle	DNA repair
Mllt1	cancer		
Mllt10	cancer		
Mllt11	cancer		

Mllt3	cancer		
Mllt4	cancer		
Mllt6	cancer		
Mlxipl		cell cycle	
Mms19			DNA repair
Mms22l			DNA repair
Mn1	cancer		
Mnat1		cell cycle	DNA repair
Mnd1		cell cycle	DNA repair
Mns1		cell cycle	
Mnt		cell cycle	
Mnx1	cancer		
Morf4l1			DNA repair
Morf4l2			DNA repair
Mos		cell cycle	
Mov10l1		cell cycle	
Mpg			DNA repair
Mpl	cancer		
Mplkip		cell cycle	
Mre11a		cell cycle	DNA repair
Mrpl41		cell cycle	
Ms4a3		cell cycle	
Msh2	cancer	cell cycle	DNA repair
Msh3		cell cycle	DNA repair
Msh4		cell cycle	DNA repair
Msh5		cell cycle	DNA repair
Msh6	cancer	cell cycle	DNA repair
Msi2	cancer		
Msn	cancer		
Msx1		cell cycle	
Msx2		cell cycle	
Mta1			DNA repair
Mta3		cell cycle	
Mtbp		cell cycle	
Mtcp1	cancer		
Mtor	cancer		DNA repair
Mtus1		cell cycle	
Muc1	cancer		
Mum1			DNA repair
Mus81			DNA repair
Mutyh	cancer		DNA repair
Myb	cancer	cell cycle	
Mybbp1a		cell cycle	
Mybl2		cell cycle	
Myc	cancer	cell cycle	
Mycl	cancer		
Mycn	cancer		
Myd88	cancer		

Myh10		cell cycle		
Myh11	cancer			
Myh9	cancer	cell cycle		
Mylk2		cell cycle		
Myo5a	cancer			
Myocd		cell cycle		
Myod1	cancer			
Myog		cell cycle		
Mzt1		cell cycle		
Naa50		cell cycle		
Nab2	cancer			
Nabp1		cell cycle	DNA repair	
Nabp2		cell cycle	DNA repair	
Nacc2		cell cycle		
Nae1		cell cycle		
Nanog		cell cycle		
Nanos2		cell cycle		
Nanos3		cell cycle		
Nasp		cell cycle		
Nbn	cancer	cell cycle	DNA repair	
Ncapd2		cell cycle		
Ncapd3		cell cycle		
Ncapg		cell cycle		
Ncapg2		cell cycle		
Ncaph		cell cycle		
Nceh1				xenobiotic metabolism
Nckipsd	cancer			
Ncoa1	cancer			
Ncoa2	cancer			
Ncoa4	cancer			
Ncor1	cancer	cell cycle		
Ncor2	cancer			
Ndc1		cell cycle		
Ndc80		cell cycle		
Nde1		cell cycle		
Ndel1		cell cycle		
Ndnl2			DNA repair	
Ndrg1	cancer	cell cycle		
Nedd1		cell cycle		
Nedd9		cell cycle		
Neil1			DNA repair	
Neil2			DNA repair	
Neil3			DNA repair	
Nek1		cell cycle		
Nek11		cell cycle		
Nek2		cell cycle		
Nek3		cell cycle		
Nek4		cell cycle		

Nek6		cell cycle		
Nek9		cell cycle		
Nell1		cell cycle		
Neurod1		cell cycle		
Neurog1		cell cycle		
Nf1	cancer			
Nf2	cancer			
Nfatc1		cell cycle		
Nfatc2	cancer			
Nfe2l2	cancer			
Nfib	cancer			
Nfkb2	cancer			
Nfkbie	cancer			
Nfrkb			DNA repair	
Nhej1			DNA repair	
Nin	cancer			
Nipbl		cell cycle		
Nkx2-1	cancer			
Nkx3-1		cell cycle		
Nle1		cell cycle		
Nlrc4		cell cycle		
Nme6		cell cycle		
Nol9		cell cycle		
Nono	cancer		DNA repair	
Notch1	cancer			
Notch2	cancer	cell cycle		
Npas2			DNA repair	
Npat		cell cycle		
Npm1	cancer	cell cycle	DNA repair	
Npm2		cell cycle		
Nppc		cell cycle		
Npr2		cell cycle		
Nr1i2				xenobiotic metabolism
Nr2c2		cell cycle		
Nr2e1		cell cycle		
Nr2f2		cell cycle		
Nr4a1		cell cycle		
Nr4a3	cancer	cell cycle		
Nras	cancer	cell cycle		
Nrg1	cancer			
Nsd1	cancer			
Nsl1		cell cycle		
Nsmce1			DNA repair	
Nsmce2		cell cycle	DNA repair	
Nsun2		cell cycle		
Nt5c2	cancer			
Nthl1			DNA repair	
Ntmt1		cell cycle		

Ntrk1	cancer		
Ntrk3	cancer		
Nubp1		cell cycle	
Nucks1			DNA repair
Nudc		cell cycle	
Nudt1			DNA repair
Nudt16		cell cycle	DNA repair
Nuf2		cell cycle	
Numa1	cancer	cell cycle	
Nup153		cell cycle	
Nup214	cancer	cell cycle	
Nup37		cell cycle	
Nup43		cell cycle	
Nup62		cell cycle	
Nup88		cell cycle	
Nup98	cancer		
Nupr1		cell cycle	
Nupr1l		cell cycle	
Nusap1		cell cycle	
Nutm1	cancer		
Nutm2	cancer		
Ofd1		cell cycle	
Ogg1			DNA repair
Oip5		cell cycle	
Olig2	cancer		
Omd	cancer		
Opn1mw		cell cycle	
Oraov1		cell cycle	DNA repair
Orc1		cell cycle	
Orc3		cell cycle	
Osm		cell cycle	
Otub1			DNA repair
Ovol1		cell cycle	
Pafah1b1		cell cycle	
Pafah1b2	cancer		
Pagr1a		cell cycle	
Pak2		cell cycle	
Pak4		cell cycle	
Palb2	cancer		DNA repair
Papd5		cell cycle	
Papd7		cell cycle	
Pard3		cell cycle	
Pard3b		cell cycle	
Pard6a		cell cycle	
Pard6b		cell cycle	
Pard6g		cell cycle	
Parg			DNA repair
Parp1			DNA repair

Parp2			DNA repair
Parp3		cell cycle	DNA repair
Parp9			DNA repair
Parpbp			DNA repair
Pax3	cancer		
Pax5	cancer		
Pax6		cell cycle	
Pax7	cancer		
Pax8	cancer		
Paxip1		cell cycle	DNA repair
Pbrm1	cancer		
Pbx1	cancer	cell cycle	
Pcid2		cell cycle	
Pcm1	cancer		
Pcna		cell cycle	DNA repair
Pcnp		cell cycle	
Pcnt		cell cycle	
Pcsk7	cancer		
Pdcd1lg2	cancer		
Pdcd2l		cell cycle	
Pdcd6ip		cell cycle	
Pde3a		cell cycle	
Pde4dip	cancer		
Pdgfb	cancer	cell cycle	
Pdgfrb	cancer	cell cycle	
Pdpn		cell cycle	
Pds5a		cell cycle	DNA repair
Pds5b		cell cycle	DNA repair
Pea15a		cell cycle	
Pebp1		cell cycle	
Pelo		cell cycle	
Per1	cancer		
Per2		cell cycle	
Pes1		cell cycle	
Pggt1b		cell cycle	
Phactr4		cell cycle	
Phb2		cell cycle	
Phf13		cell cycle	
Phf6	cancer		
Phf8		cell cycle	
Phgdh		cell cycle	
Phip		cell cycle	
Phox2b	cancer		
Pias1		cell cycle	
Pibf1		cell cycle	
Picalm	cancer		
Pid1		cell cycle	
Pidd1		cell cycle	

Pif1			DNA repair
Pik3c3		cell cycle	
Pik3ca	cancer		
Pik3r1	cancer		
Pik3r4		cell cycle	
Pim1	cancer	cell cycle	
Pim2		cell cycle	
Pim3		cell cycle	
Pin1		cell cycle	
Pinx1		cell cycle	
Piwil1		cell cycle	
Piwil2		cell cycle	
Piwil4		cell cycle	
Pkd1		cell cycle	
Pkd2		cell cycle	
Pkhd1		cell cycle	
Pkia		cell cycle	
Pkmyt1		cell cycle	
Pkn2		cell cycle	
Pkp4		cell cycle	
Pla2g16		cell cycle	
Plag1	cancer		
Plcb1		cell cycle	
Plcg1	cancer		
Pld6		cell cycle	
Plk1		cell cycle	
Plk2		cell cycle	
Plk3		cell cycle	
Plk4		cell cycle	
Plk5		cell cycle	
Plrg1		cell cycle	
Pmf1		cell cycle	
Pml	cancer	cell cycle	DNA repair
Pmp22		cell cycle	
Pms1	cancer		DNA repair
Pms2	cancer	cell cycle	DNA repair
Pnkp			DNA repair
Pnp			DNA repair
Pnpt1		cell cycle	
Poc1a		cell cycle	
Poc5		cell cycle	
Pogz		cell cycle	
Pola1			DNA repair
Polb			DNA repair
Pold1			DNA repair
Pold3			DNA repair
Pold4			DNA repair
Poldip2		cell cycle	

Pole	cancer	cell cycle	DNA repair	
Pole2			DNA repair	
Polg2			DNA repair	
Polh			DNA repair	
Poli			DNA repair	
Polk			DNA repair	
Poll			DNA repair	
Polm			DNA repair	
Poln			DNA repair	
Polq			DNA repair	
Polr2i			DNA repair	
Pomk		cell cycle		
Pon3				xenobiotic metabolism
Pot1a	cancer			
Pot1b	cancer			
Pou2af1	cancer			
Pou4f1		cell cycle		
Pou5f1	cancer			
Pparg	cancer			
Ppfibp1	cancer			
Ppm1d		cell cycle		
Ppm1g		cell cycle		
Ppp1ca		cell cycle		
Ppp1cb		cell cycle		
Ppp1r12a		cell cycle		
Ppp1r13b		cell cycle		
Ppp1r1c		cell cycle		
Ppp2ca		cell cycle		
Ppp2r1a	cancer	cell cycle		
Ppp2r2a		cell cycle		
Ppp2r2b		cell cycle		
Ppp2r2c		cell cycle		
Ppp2r2d		cell cycle		
Ppp2r3d		cell cycle		
Ppp2r4		cell cycle		
Ppp2r5b		cell cycle		
Ppp3ca		cell cycle		
Ppp4c			DNA repair	
Ppp4r2			DNA repair	
Ppp6c	cancer	cell cycle		
Prc1		cell cycle		
Prcc	cancer	cell cycle		
Prdm1	cancer			
Prdm16	cancer			
Prdm5		cell cycle		
Prdm9		cell cycle		
Prf1	cancer			
Primpol			DNA repair	

Prkaca	cancer	cell cycle	
Prkacb		cell cycle	
Prkar1a	cancer		
Prkca		cell cycle	
Prkcd		cell cycle	
Prkce		cell cycle	
Prkcg			DNA repair
Prkcq		cell cycle	
Prkdc		cell cycle	DNA repair
Prmt2		cell cycle	
Prmt6			DNA repair
Prox1		cell cycle	
Prpf19		cell cycle	DNA repair
Prpf40a		cell cycle	
Prr11		cell cycle	
Prr5		cell cycle	
Prrx1	cancer		
Psip1	cancer		
Psmc3ip		cell cycle	
Psmd10		cell cycle	
Psmd13		cell cycle	
Psmd14			DNA repair
Psme1		cell cycle	
Psme2		cell cycle	
Psme3		cell cycle	
Psme4			DNA repair
Psmg2		cell cycle	
Psrc1		cell cycle	
Pstpip1		cell cycle	
Ptch1	cancer	cell cycle	
Pten	cancer	cell cycle	
Ptgs2		cell cycle	
Ptk6	cancer		
Ptp4a1		cell cycle	
Ptpn11	cancer	cell cycle	
Ptpn13	cancer		
Ptpn3		cell cycle	
Ptpn6		cell cycle	
Ptprb	cancer		
Ptprc	cancer	cell cycle	
Ptprk	cancer	cell cycle	
Ptprv		cell cycle	
Pttg1		cell cycle	DNA repair
Pum1		cell cycle	
Pura		cell cycle	
Pwwp2a	cancer		
Rab11a		cell cycle	
Rab11fip3		cell cycle	

Rab11fip4		cell cycle	
Rab35		cell cycle	
Rabep1	cancer		
Rabgap1		cell cycle	
Rac1	cancer		
Racgap1		cell cycle	
Rack1		cell cycle	
Rad1		cell cycle	DNA repair
Rad17		cell cycle	DNA repair
Rad18			DNA repair
Rad21	cancer	cell cycle	DNA repair
Rad21l		cell cycle	DNA repair
Rad23a			DNA repair
Rad23b			DNA repair
Rad50		cell cycle	DNA repair
Rad51		cell cycle	DNA repair
Rad51ap1			DNA repair
Rad51b	cancer	cell cycle	DNA repair
Rad51c		cell cycle	DNA repair
Rad51d		cell cycle	DNA repair
Rad52			DNA repair
Rad54b			DNA repair
Rad54l			DNA repair
Rad9a		cell cycle	DNA repair
Rad9b		cell cycle	DNA repair
Raf1	cancer		
Rala		cell cycle	
Ralb		cell cycle	
Ralbp1		cell cycle	
Ralgds	cancer		
Ran		cell cycle	
Ranbp1		cell cycle	
Ranbp17	cancer		
Ranbp2	cancer		
Rap1gds1	cancer		
Rara	cancer	cell cycle	
Rassf1		cell cycle	
Rassf2		cell cycle	
Rassf4		cell cycle	
Rb1	cancer	cell cycle	
Rb1cc1		cell cycle	
Rbbp4		cell cycle	
Rbbp8		cell cycle	DNA repair
Rbl1		cell cycle	
Rbl2		cell cycle	
Rbm10	cancer		
Rbm14		cell cycle	
Rbm15	cancer		

Rbm38		cell cycle	
Rbm7		cell cycle	
Rbx1			DNA repair
Rcbtb1		cell cycle	
Rcc1		cell cycle	
Rcc2		cell cycle	
Rdx		cell cycle	
Rec114		cell cycle	
Rec8		cell cycle	DNA repair
Recql			DNA repair
Recql4	cancer	cell cycle	DNA repair
Recql5		cell cycle	DNA repair
Reep3		cell cycle	
Reep4		cell cycle	
Rel	cancer		
Ret	cancer		
Rev1			DNA repair
Rev3l			DNA repair
Rfc1			DNA repair
Rfwd3		cell cycle	DNA repair
Rgcc		cell cycle	
Rgs14		cell cycle	
Rgs2		cell cycle	
Rhno1		cell cycle	
Rhoa		cell cycle	
Rhob		cell cycle	
Rhoc		cell cycle	
Rhoh	cancer		
Rhou		cell cycle	
Rif1		cell cycle	DNA repair
Rint1		cell cycle	
Rmi2	cancer		
Rnaseh2a			DNA repair
Rnaseh2b		cell cycle	
Rnf112		cell cycle	
Rnf138			DNA repair
Rnf167		cell cycle	
Rnf168			DNA repair
Rnf169			DNA repair
Rnf2		cell cycle	
Rnf212		cell cycle	
Rnf213	cancer		
Rnf4		cell cycle	
Rnf43	cancer		
Rnf8		cell cycle	DNA repair
Rny1		cell cycle	
Rny3		cell cycle	
Rock2		cell cycle	

Rora				xenobiotic metabolism
Rorc				xenobiotic metabolism
Ros1	cancer			
Rpa1		cell cycle	DNA repair	
Rpa2		cell cycle	DNA repair	
Rpa3		cell cycle	DNA repair	
Rpain			DNA repair	
Rpl17		cell cycle		
Rpl22	cancer			
Rpl24		cell cycle		
Rpl5	cancer			
Rpn1	cancer			
Rprd1b		cell cycle		
Rprm		cell cycle		
Rps15a		cell cycle		
Rps27l		cell cycle	DNA repair	
Rps3		cell cycle	DNA repair	
Rps6		cell cycle		
Rps6ka2		cell cycle		
Rps6ka3		cell cycle		
Rps6kb1		cell cycle		
Rrm2b			DNA repair	
Rrp8		cell cycle		
Rrs1		cell cycle		
Rsph1		cell cycle		
Rspo1		cell cycle		
Rspo2	cancer			
Rspo3	cancer			
Rtel1		cell cycle	DNA repair	
Rtfdc1		cell cycle		
Runx1	cancer			
Runx1t1	cancer			
Runx3		cell cycle		
Ruvbl1		cell cycle	DNA repair	
Ruvbl2			DNA repair	
Rxfp3		cell cycle		
Sac3d1		cell cycle		
Sae1		cell cycle		
Sass6		cell cycle		
Sbds	cancer	cell cycle		
Scrib		cell cycle		
Sdc4	cancer			
Sdcbp		cell cycle		
Sdccag3		cell cycle		
Sdha	cancer			
Sdhaf2	cancer			
Sdhb	cancer			
Sdhc	cancer			

Seh1l		cell cycle	
Senp2		cell cycle	
Senp5		cell cycle	
Senp6		cell cycle	
Sept1		cell cycle	
Sept10		cell cycle	
Sept11		cell cycle	
Sept14		cell cycle	
Sept2		cell cycle	
Sept3		cell cycle	
Sept4		cell cycle	
Sept5	cancer	cell cycle	
Sept6		cell cycle	
Sept7		cell cycle	
Sept9	cancer	cell cycle	
Set	cancer		
Setbp1	cancer		
Setd2	cancer		DNA repair
Setdb2		cell cycle	
Setx			DNA repair
Sf3b1	cancer		
Sfn		cell cycle	
Sfpq	cancer	cell cycle	DNA repair
Sfr1			DNA repair
Sfrp1		cell cycle	
Sgk1		cell cycle	
Sgol1		cell cycle	
Sgol2a		cell cycle	
Sgsm3		cell cycle	
Sh2b1		cell cycle	
Sh2b3	cancer		
Sh3gl1	cancer		
Sh3glb1		cell cycle	
Shb		cell cycle	
Shfm1			DNA repair
Shprh			DNA repair
Siah1a		cell cycle	
Siah2		cell cycle	
Sik1		cell cycle	
Sin3a		cell cycle	
Sipa1		cell cycle	
Sirt1		cell cycle	DNA repair
Sirt2		cell cycle	
Sirt6			DNA repair
Sirt7		cell cycle	
Six3		cell cycle	
Ska1		cell cycle	
Ska2		cell cycle	

Ska3		cell cycle		
Skil		cell cycle		
Skp2		cell cycle		
Slbp		cell cycle		
Slc25a33		cell cycle		
Slc26a8		cell cycle		
Slc34a2	cancer			
Slc45a3	cancer			
Slc6a4		cell cycle		
Slc9a3r1		cell cycle		
Slco1a6				xenobiotic metabolism
Slf1		cell cycle	DNA repair	
Slfn1		cell cycle		
Slx1b			DNA repair	
Slx4		cell cycle	DNA repair	
Smad2	cancer			
Smad3	cancer	cell cycle		
Smad4	cancer			
Smarca4	cancer			
Smarca5			DNA repair	
Smarcad1		cell cycle	DNA repair	
Smarcb1	cancer	cell cycle	DNA repair	
Smarcd1	cancer			
Smarcd3		cell cycle		
Smarce1	cancer			
Smc1a		cell cycle	DNA repair	
Smc1b		cell cycle		
Smc2		cell cycle		
Smc3		cell cycle	DNA repair	
Smc4		cell cycle		
Smc5		cell cycle	DNA repair	
Smc6			DNA repair	
Smg1			DNA repair	
Smo	cancer			
Smpd3		cell cycle		
Smug1			DNA repair	
Snd1	cancer			
Snx18		cell cycle		
Snx33		cell cycle		
Snx9		cell cycle		
Socs1	cancer			
Son		cell cycle		
Sox15		cell cycle		
Sox2	cancer	cell cycle		
Sox4		cell cycle		
Sox9		cell cycle		
Spag5		cell cycle		
Spag8		cell cycle		

Spast		cell cycle	
Spata22		cell cycle	DNA repair
Spc24		cell cycle	
Spc25		cell cycle	
Spdl1		cell cycle	
Spdya		cell cycle	
Spdye4a		cell cycle	
Specc1	cancer		
Specc1l		cell cycle	
Spen	cancer		
Spg20		cell cycle	
Sphk1		cell cycle	
Spice1		cell cycle	
Spidr			DNA repair
Spin1		cell cycle	
Spin2c		cell cycle	
Spire1		cell cycle	
Spire2		cell cycle	
Spo11		cell cycle	
Spop	cancer		
Sprtn			DNA repair
Spry1		cell cycle	
Spry2		cell cycle	
Sptbn1		cell cycle	
Src		cell cycle	
Srgap3	cancer		
Srpk2		cell cycle	
Srsf2	cancer		
Srsf3	cancer		
Ss18	cancer		
Ss18l1	cancer		
Ssrp1			DNA repair
Sstr5		cell cycle	
Ssx9	cancer		
Ssxa1	cancer		
Ssxb1	cancer		
Ssxb10	cancer		
Ssxb2	cancer		
Ssxb3	cancer		
Ssxb5	cancer		
Ssxb6	cancer		
Ssxb8	cancer		
Ssxb9	cancer		
Stag1		cell cycle	
Stag2	cancer	cell cycle	
Stag3		cell cycle	
Stambp		cell cycle	
Stard13		cell cycle	

Stard9		cell cycle		
Stat3	cancer	cell cycle		
Stat5a		cell cycle		
Stat5b	cancer	cell cycle		
Stat6	cancer			
Steap3		cell cycle		
Stil	cancer	cell cycle		
Stk10		cell cycle		
Stk11	cancer	cell cycle		
Stk24		cell cycle		
Stk26		cell cycle		
Stmn1		cell cycle		
Stox1		cell cycle		
Stra13		cell cycle	DNA repair	
Stra8		cell cycle		
Strada		cell cycle		
Stradb		cell cycle		
Strn	cancer			
Stub1			DNA repair	
Stxbp4		cell cycle		
Sufu	cancer			
Sult1a1				xenobiotic metabolism
Sult1b1				xenobiotic metabolism
Sun1		cell cycle		
Supt16			DNA repair	
Susd2		cell cycle		
Suv39h1		cell cycle		
Suv39h2		cell cycle		
Suz12	cancer			
Swi5			DNA repair	
Swsap1			DNA repair	
Syce1		cell cycle		
Syce1l		cell cycle		
Syce2		cell cycle		
Syce3		cell cycle		
Sycp1		cell cycle	DNA repair	
Sycp2		cell cycle		
Sycp3		cell cycle	DNA repair	
Syde1		cell cycle		
Syf2		cell cycle		
Syk	cancer			
Syne4		cell cycle		
Tacc1		cell cycle		
Tacc2		cell cycle		
Tacc3		cell cycle		
Tada2a		cell cycle		
Tada3		cell cycle		
Taf1		cell cycle		

Taf10		cell cycle	
Taf15	cancer		
Taf2		cell cycle	
Taf6		cell cycle	
Tal1	cancer	cell cycle	
Tal2	cancer		
Taok1		cell cycle	DNA repair
Taok2		cell cycle	
Taok3		cell cycle	DNA repair
Tardbp		cell cycle	
Tas1r2		cell cycle	
Tas2r121		cell cycle	
Tbl1xr1	cancer		
Tbrg1		cell cycle	
Tbx3	cancer	cell cycle	
Tcea1	cancer		
Tcf12	cancer		
Tcf19		cell cycle	
Tcf3	cancer		
Tcf7l1		cell cycle	
Tcf7l2	cancer	cell cycle	
Tcl1	cancer		
Tdg			DNA repair
Tdp1			DNA repair
Tdp2			DNA repair
Tdpoz1	cancer		
Tdpoz2	cancer		
Tdpoz3	cancer		
Tdpoz4	cancer		
Tdpoz5	cancer		
Tdrd1		cell cycle	
Tdrd12		cell cycle	
Tdrd9		cell cycle	
Tdrkh		cell cycle	
Terf1		cell cycle	
Terf2		cell cycle	
Terf2ip			DNA repair
Tert	cancer	cell cycle	
Tet1	cancer		
Tet2	cancer	cell cycle	
Tex11		cell cycle	
Tex12		cell cycle	DNA repair
Tex14		cell cycle	
Tex15		cell cycle	DNA repair
Tex19.1		cell cycle	
Tex19.2		cell cycle	
Tex24		cell cycle	
Tex40		cell cycle	

Tfap4		cell cycle	
Tfdp1		cell cycle	
Tfdp2		cell cycle	
Tfe3	cancer		
Tfeb	cancer		
Tfg	cancer		
Tfpt	cancer		DNA repair
Tfrc	cancer		
Tgfa		cell cycle	
Tgfb1		cell cycle	
Tgfb2		cell cycle	
Tgfbr2	cancer		
Tgm1		cell cycle	
Thap1		cell cycle	
Thbs1		cell cycle	
Thoc1		cell cycle	
Thoc5		cell cycle	
Thrap3	cancer		
Ticrr		cell cycle	DNA repair
Tigar			DNA repair
Timeless		cell cycle	
Timp2		cell cycle	
Tipin		cell cycle	
Tiprl		cell cycle	
Tjp3		cell cycle	
Tlk1		cell cycle	
Tlk2		cell cycle	
Tlx1	cancer		
Tlx3	cancer		
Tmem161a			DNA repair
Tmem67		cell cycle	
Tmod3		cell cycle	
Tmprss11a		cell cycle	
Tmprss2	cancer		
Tnf		cell cycle	
Tnfaip3	cancer	cell cycle	
Tnfrsf14	cancer		
Tnfrsf17	cancer		
Tnks		cell cycle	
Tom1l1		cell cycle	
Tom1l2		cell cycle	
Tonsl			DNA repair
Top1	cancer		
Top2a		cell cycle	
Top2b		cell cycle	
Topaz1		cell cycle	
Topbp1		cell cycle	DNA repair
Tpd52l1		cell cycle	

Tpm4	cancer		
Tpr	cancer	cell cycle	
Tpra1		cell cycle	
Tpx2		cell cycle	
Traf7	cancer		
Trex2			DNA repair
Trim21		cell cycle	
Trim24	cancer		
Trim27	cancer		
Trim28			DNA repair
Trim32		cell cycle	
Trim33	cancer		
Trim35		cell cycle	
Trim36		cell cycle	
Trim37		cell cycle	
Trim71		cell cycle	
Triobp		cell cycle	
Trip11	cancer		
Trip12			DNA repair
Trip13		cell cycle	DNA repair
Trnp1		cell cycle	
Trp53	cancer	cell cycle	DNA repair
Trp53bp1		cell cycle	DNA repair
Trp53bp2		cell cycle	
Trp53inp1		cell cycle	
Trp63		cell cycle	
Trp73		cell cycle	
Trpc2			DNA repair
Trrap	cancer	cell cycle	DNA repair
Tsc1	cancer	cell cycle	
Tsc2	cancer	cell cycle	
Tsg101		cell cycle	
Tshr	cancer		
Tspyl2		cell cycle	
Ttc28		cell cycle	
Ttc5			DNA repair
Ttk		cell cycle	
Ttl	cancer		
Ttyh1		cell cycle	
Tubb1		cell cycle	
Tubb3		cell cycle	
Tubb5		cell cycle	
Tubg1		cell cycle	
Tubgcp2		cell cycle	
Tubgcp3		cell cycle	
Tubgcp4		cell cycle	
Tubgcp5		cell cycle	
Tubgcp6		cell cycle	

Tunar		cell cycle		
Twist1			DNA repair	
Txlng		cell cycle		
Txnip		cell cycle		
Txnl4b		cell cycle		
U2af1	cancer			
Uba3		cell cycle		
Ubb		cell cycle		
Ube2a			DNA repair	
Ube2b		cell cycle	DNA repair	
Ube2c		cell cycle		
Ube2d3			DNA repair	
Ube2e2		cell cycle		
Ube2f			DNA repair	
Ube2i		cell cycle		
Ube2l3		cell cycle		
Ube2n			DNA repair	
Ube2s		cell cycle		
Ube2t			DNA repair	
Ube2u			DNA repair	
Ube2v1			DNA repair	
Ube2v2			DNA repair	
Ube2w			DNA repair	
Ubr2		cell cycle		
Ubr5	cancer		DNA repair	
Uchl5			DNA repair	
Uckl1		cell cycle		
Ugt1a1				xenobiotic metabolism
Ugt1a10				xenobiotic metabolism
Ugt1a2				xenobiotic metabolism
Ugt1a5				xenobiotic metabolism
Ugt1a6a				xenobiotic metabolism
Ugt1a6b				xenobiotic metabolism
Ugt1a7c				xenobiotic metabolism
Ugt1a8				xenobiotic metabolism
Ugt1a9				xenobiotic metabolism
Ugt2b1				xenobiotic metabolism
Uhmk1		cell cycle		
Uhrf1		cell cycle	DNA repair	
Uhrf2		cell cycle		
Uimc1		cell cycle	DNA repair	
Ung			DNA repair	
Upf1		cell cycle	DNA repair	
Urgcp		cell cycle		
Ush1c		cell cycle		
Usp1			DNA repair	
Usp10			DNA repair	
Usp16		cell cycle		

Usp19		cell cycle	
Usp2		cell cycle	
Usp22		cell cycle	
Usp28		cell cycle	DNA repair
Usp3		cell cycle	DNA repair
Usp32	cancer		
Usp33		cell cycle	
Usp37		cell cycle	
Usp39		cell cycle	
Usp44		cell cycle	
Usp45			DNA repair
Usp47		cell cycle	DNA repair
Usp7			DNA repair
Usp8	cancer	cell cycle	
Usp9x		cell cycle	
Utp14b		cell cycle	
Uvrag		cell cycle	DNA repair
Uvssa			DNA repair
Vash1		cell cycle	
Vcp			DNA repair
Vcpip1		cell cycle	
Vps4a		cell cycle	
Vps4b		cell cycle	
Vrk1		cell cycle	
Vti1a	cancer		
Wac		cell cycle	
Wapl		cell cycle	
Was	cancer		
Wash1		cell cycle	
Wasl		cell cycle	
Wdr43		cell cycle	
Wdr48			DNA repair
Wdr6		cell cycle	
Wdr62		cell cycle	
Wdr76		cell cycle	
Wdr81		cell cycle	
Wee1		cell cycle	
Wee2		cell cycle	
Wfs1		cell cycle	
Whsc1	cancer		DNA repair
Whsc1l1	cancer		
Wif1	cancer		
Wiz		cell cycle	
Wnt10b		cell cycle	
Wnt4		cell cycle	
Wnt5a		cell cycle	
Wnt9a		cell cycle	
Wrap73		cell cycle	

Wrn	cancer		DNA repair
Wrnip1			DNA repair
Wt1	cancer		
Wtap		cell cycle	
Wwtr1	cancer		
Xab2			DNA repair
Xpa	cancer		DNA repair
Xpc	cancer	cell cycle	DNA repair
Xpo1	cancer	cell cycle	
Xrcc1			DNA repair
Xrcc2		cell cycle	DNA repair
Xrcc3		cell cycle	DNA repair
Xrcc4			DNA repair
Xrcc5			DNA repair
Xrcc6			DNA repair
Xrn1		cell cycle	
Xrn2			DNA repair
Ywhae	cancer		
Ywhah		cell cycle	
Yy1			DNA repair
Zak		cell cycle	
Zbtb1			DNA repair
Zbtb16	cancer		
Zbtb49		cell cycle	
Zc3hc1		cell cycle	
Zcchc8	cancer		
Zfhx3	cancer		
Zfp207		cell cycle	
Zfp318		cell cycle	
Zfp365		cell cycle	
Zfp369		cell cycle	
Zfp384	cancer		
Zfp385a		cell cycle	
Zfp386		cell cycle	
Zfp503		cell cycle	
Zfp521	cancer		
Zfp655		cell cycle	
Zfp703		cell cycle	
Zfp830		cell cycle	
Zfyve19		cell cycle	
Zfyve26		cell cycle	DNA repair
Znhit1		cell cycle	
Zpr1		cell cycle	
Zranb3			DNA repair
Zrsr1	cancer		
Zrsr2	cancer		
Zswim7			DNA repair
Zw10		cell cycle	

Zwilch		cell cycle		
Zwint		cell cycle		
**Grand Total**	**641**	**1432**	**407**	**45**

Supplemental Table S3. CNVs called by aCGH and comparison to CNVnator (NGS coverage-based algorithm) calls.

chromosome	start of R-gada CNV call (mm10 coordinates)	end of R-gada CNV call (mm10 coordinates)	size	name	# CNVs called by CNVnator in region	CNV Classification Type
chr1	8,977,823	9,051,197	73,374	CNV1	1	Simple
chr1	11,245,663	11,269,836	24,173	CNV2	1	Simple
chr1	11,703,347	11,918,632	215,285	CNV3	10	Complex
chr1	11,924,866	11,931,700	6,834	CNV4	1	Simple
chr1	14,883,606	14,896,597	12,991	CNV5	1	Simple
chr1	20,335,207	20,374,697	39,490	CNV6	0	Simple
chr1	20,504,760	20,562,015	57,255	CNV7	1	Simple
chr1	29,112,070	29,148,585	36,515	CNV8	2	Complex
chr1	48,675,149	48,707,315	32,166	CNV9	5	Complex
chr1	59,814,900	59,821,568	6,668	CNV10	1	Simple
chr1	61,744,707	61,752,097	7,390	CNV11	1	Simple
chr1	68,199,570	68,250,365	50,795	CNV12	1	Simple
chr1	88,209,053	88,317,518	108,465	CNV13	2	Complex
chr1	115,903,193	115,913,905	10,712	CNV14	0	Simple
chr1	116,332,959	116,350,366	17,407	CNV15	1	Simple
chr1	164,025,139	164,058,471	33,332	CNV16	2	Complex

chr1	171,504,889	171,575,973	71,084	CNV17	4	Complex
chr1	184,979,019	185,396,837	417,818	CNV18	4	Complex
chr2	24,935,959	24,981,137	45,178	CNV19	0	Simple
chr2	31,192,773	31,203,334	10,561	CNV20	1	Simple
chr2	41,028,946	41,038,306	9,360	CNV21	0	Simple
chr2	46,371,360	46,548,734	177,374	CNV22	1	Simple
chr2	50,849,618	50,908,707	59,089	CNV23	0	Simple
chr2	51,607,290	52,204,431	597,141	CNV24	15	Complex
chr2	83,816,806	83,905,314	88,508	CNV25	7	Complex
chr2	85,815,643	85,821,297	5,654	CNV26	0	Simple
chr2	85,901,465	85,909,798	8,333	CNV27	1	Simple
chr2	86,132,070	86,144,930	12,860	CNV28	1	Simple
chr2	86,385,227	86,512,531	127,304	CNV29	6	Complex
chr2	87,072,607	87,110,411	37,804	CNV30	0	Simple
chr2	87,402,071	87,417,831	15,760	CNV31	3	Complex
chr2	88,395,883	88,402,255	6,372	CNV32	0	Simple
chr2	88,866,285	88,893,657	27,372	CNV33	0	Simple
chr2	121,474,126	121,490,316	16,190	CNV34	1	Simple
chr2	128,606,503	128,999,857	393,354	CNV35	7	Complex

chr2	129,206,701	129,225,065	18,364	CNV36	0	Simple
chr2	139,907,500	139,918,156	10,656	CNV37	4	Complex
chr2	141,845,357	141,877,747	32,390	CNV38	1	Simple
chr2	143,691,607	143,707,446	15,839	CNV39	1	Simple
chr2	146,018,411	146,198,098	179,687	CNV40	12	Complex
chr2	146,967,957	146,977,748	9,791	CNV41	0	Simple
chr2	147,655,650	147,706,977	51,327	CNV42	3	Complex
chr2	149,030,281	149,078,332	48,051	CNV43	3	Complex
chr2	153,807,199	154,018,882	211,683	CNV44	6	Complex
chr2	154,119,082	154,680,452	561,370	CNV45	10	Complex
chr2	156,917,407	156,936,288	18,881	CNV46	0	Simple
chr2	161,710,942	162,231,116	520,174	CNV47	18	Complex
chr3	7,492,129	7,538,999	46,870	CNV48	0	Simple
chr3	25,051,626	25,064,316	12,690	CNV50	0	Simple
chr3	37,972,568	38,435,326	462,758	CNV51	4	Complex
chr3	47,958,634	48,014,515	55,881	CNV52	9	Complex
chr3	49,355,885	49,456,870	100,985	CNV53	8	Complex
chr3	70,661,260	70,750,573	89,313	CNV54	14	Complex
chr3	72,888,214	72,899,900	11,686	CNV55	1	Simple

chr3	76,250,459	76,263,597	13,138	CNV56	0	Simple
chr3	79,042,658	79,057,176	14,518	CNV57	0	Simple
chr3	79,211,728	79,230,700	18,972	CNV58	0	Simple
chr3	81,108,183	81,124,245	16,062	CNV59	1	Simple
chr3	81,186,730	81,195,719	8,989	CNV60	0	Simple
chr3	82,522,808	82,537,867	15,059	CNV61	1	Simple
chr3	87,127,659	87,527,598	399,939	CNV62	10	Complex
chr3	88,778,677	88,827,583	48,906	CNV63	10	Complex
chr3	94,665,658	94,696,424	30,766	CNV64	4	Complex
chr3	105,846,749	105,854,282	7,533	CNV65	0	Simple
chr3	106,123,095	106,307,629	184,534	CNV66	1	Simple
chr3	114,089,044	114,101,617	12,573	CNV67	0	Simple
chr3	114,675,965	114,766,505	90,540	CNV68	6	Complex
chr3	122,216,034	122,236,949	20,915	CNV69	1	Simple
chr3	138,566,672	138,582,993	16,321	CNV70	0	Simple
chr3	139,839,170	139,878,648	39,478	CNV71	0	Simple
chr4	19,986,101	20,084,711	98,610	CNV72	2	Complex
chr4	31,955,346	32,057,183	101,837	CNV73	8	Complex
chr4	32,001,415	32,108,282	106,867	CNV74	7	Complex

chr4	43,370,151	43,430,529	60,378	CNV75	0	Simple
chr4	52,947,219	52,965,064	17,845	CNV76	0	Simple
chr4	62,495,468	62,524,243	28,775	CNV77	1	Simple
chr4	74,031,944	74,037,090	5,146	CNV78	0	Simple
chr4	74,217,665	74,227,718	10,053	CNV79	1	Simple
chr4	75,990,614	76,337,386	346,772	CNV80	5	Complex
chr4	76,340,333	76,354,046	13,713	CNV81	3	Complex
chr4	76,355,918	76,584,514	228,596	CNV82	2	Complex
chr4	111,922,201	112,608,339	686,138	CNV83	43	Complex
chr4	111,952,594	112,780,093	827,499	CNV84	56	Complex
chr4	112,047,785	112,780,093	732,308	CNV85	45	Complex
chr4	112,066,910	112,780,093	713,183	CNV86	41	Complex
chr4	112,350,431	112,780,093	429,662	CNV87	29	Complex
chr4	112,559,367	113,113,016	553,649	CNV88	48	Complex
chr4	112,779,050	113,113,016	333,966	CNV89	26	Complex
chr4	113,918,068	114,199,039	280,971	CNV90	69	Complex
chr4	118,098,652	118,109,631	10,979	CNV91	0	Simple
chr4	118,585,173	118,605,722	20,549	CNV92	2	Complex
chr4	133,155,079	133,164,522	9,443	CNV94	0	Simple

chr4	133,930,003	133,955,533	25,530	CNV95	2	Complex
chr4	135,649,234	135,671,995	22,761	CNV96	2	Complex
chr4	136,057,736	136,074,376	16,640	CNV97	2	Complex
chr4	141,392,413	141,440,480	48,067	CNV98	0	Simple
chr4	143,289,486	143,310,088	20,602	CNV99	0	Simple
chr4	143,666,881	143,842,436	175,555	CNV100	30	Complex
chr4	143,669,107	143,855,451	186,344	CNV101	32	Complex
chr4	145,432,767	145,602,971	170,204	CNV102	11	Complex
chr4	148,553,835	148,562,213	8,378	CNV103	0	Simple
chr4	154,187,624	154,194,911	7,287	CNV104	0	Simple
chr4	154,861,565	154,878,580	17,015	CNV105	3	Complex
chr5	5,109,472	5,116,828	7,356	CNV106	1	Simple
chr5	6,712,172	6,745,203	33,031	CNV107	1	Simple
chr5	7,108,520	7,155,864	47,344	CNV108	5	Complex
chr5	13,793,134	13,883,147	90,013	CNV109	6	Complex
chr5	36,010,197	36,021,657	11,460	CNV110	0	Simple
chr5	37,488,373	37,507,515	19,142	CNV111	2	Complex
chr5	44,054,520	44,064,377	9,857	CNV112	1	Simple
chr5	48,806,501	48,866,367	59,866	CNV113	1	Simple

chr5	136,873,132	136,880,508	7,376	CNV114	0	Simple
chr5	138,206,709	138,233,476	26,767	CNV115	2	Complex
chr5	139,636,398	139,649,709	13,311	CNV116	0	Simple
chr6	8,091,059	8,100,960	9,901	CNV117	1	Simple
chr6	10,820,315	10,832,439	12,124	CNV118	0	Simple
chr6	23,509,757	23,520,065	10,308	CNV119	5	Complex
chr6	71,883,324	71,892,732	9,408	CNV120	0	Simple
chr6	72,023,690	72,040,459	16,769	CNV121	3	Complex
chr6	106,150,858	106,476,074	325,216	CNV122	9	Complex
chr6	106,479,044	106,493,102	14,058	CNV123	1	Simple
chr6	109,898,008	109,962,591	64,583	CNV124	1	Simple
chr6	111,302,147	111,323,141	20,994	CNV125	0	Simple
chr6	116,485,524	116,570,763	85,239	CNV126	0	Simple
chr6	125,628,849	125,892,585	263,736	CNV127	2	Complex
chr6	128,633,923	128,788,449	154,526	CNV128	6	Complex
chr6	129,634,754	129,842,393	207,639	CNV129	10	Complex
chr6	129,950,385	130,454,449	504,064	CNV130	88	Complex
chr6	146,129,516	146,154,395	24,879	CNV131	0	Simple
chr6	146,480,984	146,807,421	326,437	CNV132	18	Complex

chr7	26,568,559	26,583,184	14,625	CNV133	1	Simple
chr7	29,792,506	30,400,746	608,240	CNV134	18	Complex
chr7	36,903,218	37,459,735	556,517	CNV135	5	Complex
chr7	50,207,340	50,222,092	14,752	CNV136	0	Simple
chr7	55,061,648	55,089,474	27,826	CNV137	2	Complex
chr7	57,840,731	57,849,909	9,178	CNV138	1	Simple
chr7	60,049,636	60,075,813	26,177	CNV139	3	Complex
chr7	85,534,611	85,563,625	29,014	CNV140	1	Simple
chr7	86,682,987	86,728,513	45,526	CNV141	3	Complex
chr7	97,494,251	97,502,334	8,083	CNV142	0	Simple
chr7	98,153,447	98,159,610	6,163	CNV143	0	Simple
chr7	99,218,481	99,242,434	23,953	CNV144	0	Simple
chr7	99,798,476	99,802,744	4,268	CNV145	1	Simple
chr7	100,682,247	100,687,430	5,183	CNV146	4	Complex
chr7	102,499,902	102,521,448	21,546	CNV147	0	Simple
chr7	103,543,395	103,562,369	18,974	CNV148	1	Simple
chr7	103,696,079	103,912,126	216,047	CNV149	14	Complex
chr7	104,281,910	104,362,542	80,632	CNV150	2	Complex
chr7	104,487,277	104,548,245	60,968	CNV151	10	Complex

chr7	106,425,755	106,643,080	217,325	CNV152	12	Complex
chr7	107,105,859	107,127,206	21,347	CNV153	4	Complex
chr7	140,165,697	140,816,973	651,276	CNV154	30	Complex
chr8	16,603,319	16,623,922	20,603	CNV155	3	Complex
chr8	19,370,138	19,470,413	100,275	CNV156	14	Complex
chr8	31,734,588	31,798,279	63,691	CNV157	2	Complex
chr8	33,831,125	33,886,267	55,142	CNV158	0	Simple
chr8	35,463,754	35,470,233	6,479	CNV159	0	Simple
chr8	37,819,768	37,964,005	144,237	CNV160	9	Complex
chr8	39,242,017	39,254,963	12,946	CNV161	2	Complex
chr8	58,647,036	58,658,911	11,875	CNV162	0	Simple
chr8	63,231,258	63,295,943	64,685	CNV163	2	Complex
chr8	63,310,736	63,324,409	13,673	CNV164	1	Simple
chr8	63,354,746	63,363,146	8,400	CNV165	0	Simple
chr8	82,049,288	82,065,919	16,631	CNV166	1	Simple
chr8	83,223,232	83,237,972	14,740	CNV167	4	Complex
chr8	113,718,698	113,726,924	8,226	CNV168	0	Simple
chr8	113,783,946	113,800,505	16,559	CNV169	0	Simple
chr8	121,786,998	121,795,409	8,411	CNV170	0	Simple

chr8	124,807,261	124,821,661	14,400	CNV171	3	Complex
chr8	125,275,786	125,603,762	327,976	CNV172	3	Complex
chr8	127,164,340	127,172,123	7,783	CNV173	1	Simple
chr9	38,240,631	38,422,124	181,493	CNV174	21	Complex
chr9	73,771,421	73,780,290	8,869	CNV175	0	Simple
chr9	80,218,865	80,230,523	11,658	CNV176	0	Simple
chr9	87,395,340	87,415,334	19,994	CNV177	1	Simple
chr9	104,460,782	105,021,306	560,524	CNV178	8	Complex
chr9	105,023,496	105,033,079	9,583	CNV179	0	Simple
chr10	13,199,877	13,624,466	424,589	CNV180	7	Complex
chr10	13,763,949	13,776,687	12,738	CNV181	0	Simple
chr10	28,625,315	29,155,784	530,469	CNV182	7	Complex
chr10	67,931,697	68,074,235	142,538	CNV183	1	Simple
chr10	89,889,069	89,948,844	59,775	CNV184	2	Complex
chr10	93,610,851	93,623,145	12,294	CNV185	3	Complex
chr10	93,683,520	93,694,187	10,667	CNV186	1	Simple
chr10	105,147,044	105,672,479	525,435	CNV187	7	Complex
chr10	106,979,486	107,044,304	64,818	CNV188	0	Simple
chr10	125,258,632	125,357,630	98,998	CNV189	0	Simple

chr11	4,617,196	4,625,581	8,385	CNV190	0	Simple
chr11	28,406,148	28,423,800	17,652	CNV191	1	Simple
chr11	29,133,936	29,148,705	14,769	CNV192	0	Simple
chr11	29,322,325	29,362,878	40,553	CNV193	3	Complex
chr11	51,027,977	51,041,483	13,506	CNV194	2	Complex
chr11	54,814,006	54,824,436	10,430	CNV195	0	Simple
chr11	58,834,730	58,843,551	8,821	CNV196	0	Simple
chr11	71,170,125	71,296,816	126,691	CNV197	7	Complex
chr11	74,355,018	74,388,327	33,309	CNV198	4	Complex
chr11	87,834,135	87,861,485	27,350	CNV199	0	Simple
chr11	89,087,113	89,102,010	14,897	CNV200	1	Simple
chr11	89,389,901	89,398,763	8,862	CNV201	0	Simple
chr11	90,010,003	90,023,126	13,123	CNV202	3	Complex
chr11	90,177,799	90,258,292	80,493	CNV203	3	Complex
chr11	99,673,098	99,697,296	24,198	CNV204	3	Complex
chr11	103,509,919	103,527,931	18,012	CNV205	1	Simple
chr11	114,962,715	114,985,929	23,214	CNV206	3	Complex
chr11	116,742,646	116,765,470	22,824	CNV207	4	Complex
chr12	16,607,032	16,629,183	22,151	CNV208	0	Simple

chr12	21,192,505	21,237,815	45,310	CNV209	4	Complex
chr12	29,896,600	30,176,389	279,789	CNV210	3	Complex
chr12	35,000,989	35,531,721	530,732	CNV211	10	Complex
chr12	41,298,789	41,304,189	5,400	CNV212	0	Simple
chr12	61,017,560	61,076,252	58,692	CNV213	2	Complex
chr12	73,294,499	73,303,006	8,507	CNV214	0	Simple
chr12	100,728,952	101,123,906	394,954	CNV215	4	Complex
chr12	103,710,635	103,987,695	277,060	CNV216	13	Complex
chr12	104,347,614	104,358,767	11,153	CNV217	1	Simple
chr12	113,437,603	113,480,579	42,976	CNV218	5	Complex
chr12	113,750,353	114,382,618	632,265	CNV219	72	Complex
chr12	113,796,687	114,829,882	1,033,195	CNV220	131	Complex
chr12	114,381,146	114,829,882	448,736	CNV221	63	Complex
chr12	114,384,000	115,024,811	640,811	CNV222	74	Complex
chr12	115,011,988	115,058,626	46,638	CNV223	4	Complex
chr12	115,550,979	115,809,134	258,155	CNV224	34	Complex
chr12	115,847,679	115,975,177	127,498	CNV225	20	Complex
chr12	117,717,294	117,722,867	5,573	CNV226	0	Simple
chr13	23,402,190	23,412,150	9,960	CNV227	2	Complex

chr13	67,426,402	67,437,626	11,224	CNV228	0	Simple
chr13	68,931,593	68,941,838	10,245	CNV229	2	Complex
chr13	95,458,862	95,762,089	303,227	CNV230	3	Complex
chr13	95,763,144	95,769,348	6,204	CNV231	0	Simple
chr13	95,771,337	96,032,280	260,943	CNV232	4	Complex
chr13	98,598,998	98,620,562	21,564	CNV233	0	Simple
chr13	116,488,754	116,565,169	76,415	CNV234	1	Simple
chr13	117,686,179	117,934,343	248,164	CNV235	4	Complex
chr13	119,394,499	119,495,445	100,946	CNV236	4	Complex
chr14	21,212,092	21,216,009	3,917	CNV237	3	Complex
chr14	22,543,520	22,567,910	24,390	CNV238	0	Simple
chr14	22,903,971	22,930,748	26,777	CNV239	1	Simple
chr14	49,927,316	50,036,074	108,758	CNV240	5	Complex
chr14	50,962,668	50,974,797	12,129	CNV241	4	Complex
chr14	51,379,141	51,954,511	575,370	CNV242	43	Complex
chr14	51,956,322	51,971,989	15,667	CNV243	5	Complex
chr14	60,697,938	60,707,382	9,444	CNV244	0	Simple
chr14	69,472,714	69,692,315	219,601	CNV245	5	Complex
chr14	77,231,193	77,239,297	8,104	CNV246	1	Simple

chr14	77,240,922	77,767,813	526,891	CNV247	4	Complex
chr14	88,650,421	88,702,265	51,844	CNV248	7	Complex
chr14	90,383,319	90,437,558	54,239	CNV249	4	Complex
chr14	100,516,072	100,819,089	303,017	CNV250	18	Complex
chr14	101,133,530	101,159,329	25,799	CNV251	1	Simple
chr14	109,315,613	109,440,021	124,408	CNV252	2	Complex
chr14	119,828,821	119,835,959	7,138	CNV253	5	Complex
chr14	121,670,441	121,677,892	7,451	CNV254	0	Simple
chr15	32,198,496	32,490,028	291,532	CNV255	9	Complex
chr15	39,695,068	40,003,295	308,227	CNV256	7	Complex
chr15	46,930,319	46,956,114	25,795	CNV257	0	Simple
chr15	47,795,286	47,806,194	10,908	CNV258	0	Simple
chr15	70,842,485	70,889,566	47,081	CNV259	10	Complex
chr15	78,933,950	78,944,254	10,304	CNV260	1	Simple
chr15	91,075,146	91,135,504	60,358	CNV261	8	Complex
chr16	28,613,434	28,674,852	61,418	CNV262	2	Complex
chr16	45,235,949	45,641,980	406,031	CNV263	9	Complex
chr16	59,372,305	59,763,158	390,853	CNV264	7	Complex
chr16	62,121,194	62,173,208	52,014	CNV265	3	Complex

chr16	64,975,653	64,993,703	18,050	CNV266	3	Complex
chr16	67,881,291	67,932,144	50,853	CNV267	2	Complex
chr16	87,611,354	87,624,644	13,290	CNV268	0	Simple
chr16	91,002,846	91,013,863	11,017	CNV269	0	Simple
chr16	93,624,681	93,639,559	14,878	CNV270	8	Complex
chr17	27,248,448	27,448,647	200,199	CNV271	5	Complex
chr17	36,142,310	36,227,918	85,608	CNV272	20	Complex
chr17	36,911,115	36,920,990	9,875	CNV273	0	Simple
chr17	37,941,852	37,953,919	12,067	CNV274	1	Simple
chr17	39,945,764	40,002,726	56,962	CNV275	2	Complex
chr17	40,868,431	41,085,825	217,394	CNV276	16	Complex
chr17	55,193,603	55,636,845	443,242	CNV277	24	Complex
chr17	84,910,270	84,936,764	26,494	CNV278	1	Simple
chr18	12,476,035	12,482,423	6,388	CNV279	0	Simple
chr18	31,612,672	31,637,765	25,093	CNV280	3	Complex
chr18	32,775,929	32,795,858	19,929	CNV281	5	Complex
chr18	37,324,987	37,330,588	5,601	CNV282	1	Simple
chr18	43,159,278	43,177,946	18,668	CNV283	0	Simple
chr18	62,516,177	62,525,116	8,939	CNV284	0	Simple

chr18	63,286,953	63,294,764	7,811	CNV285	0	Simple
chr18	68,121,447	68,137,425	15,978	CNV286	0	Simple
chr18	70,007,372	70,366,675	359,303	CNV287	23	Complex
chr18	75,532,126	76,195,878	663,752	CNV288	7	Complex
chr18	85,866,855	85,906,167	39,312	CNV289	7	Complex
chr19	9,152,536	9,724,688	572,152	CNV290	44	Complex
chr19	11,559,397	11,580,016	20,619	CNV291	2	Complex
chr19	22,171,199	22,181,470	10,271	CNV292	1	Simple
chr19	24,109,346	24,129,266	19,920	CNV293	0	Simple
chr19	36,910,306	36,972,609	62,303	CNV294	0	Simple
chr19	37,236,210	37,378,375	142,165	CNV295	1	Simple
chr19	48,377,745	48,397,326	19,581	CNV296	1	Simple
chr19	50,491,118	50,706,252	215,134	CNV297	3	Complex
chrX	80,532,212	80,576,971	44,759	CNV298	7	Complex
chrX	136,328,010	136,366,308	38,298	CNV299	3	Complex
chrX	158,612,813	158,643,816	31,003	CNV300	0	Simple

Supplemental Table S4. Number of variants detected after downsampling sequence data from Health Canada MutaMouse animals

Health Canada Mutamouse Animal	# Variants in Original Data	# Variants in Downsampled Data	Unique Variants in Original Data	Unique Variants in Downsampled Data	Intersection (%)
1	4,994,589	4,833,711	369,675	208,797	88.88
2	4,961,695	4,725,026	455,428	218,759	86.99
3	5,144,580	4,980,247	380,280	215,947	88.88
4	4,949,501	4,789,925	367,857	208,281	88.83
5	5,161,277	4,947,631	423,310	209,664	88.21

The original sequence data for the Covance and Health Canada samples differed significantly in terms of coverage, with the Covance samples having a median depth of 28-32x and the Health Canada samples having a median depth of 54-81x. To investigate the effect that read depth had on the variants called in the animals from these two colonies, the Health Canada data was downsampled to be comparable to the Covance data using the Picard DownsampleSam tool. The resulting downsampled data had a median depth of 28x, 28x, 28x, 27x and 29x, respectively. The same analysis described in the Variant Calling section of the Methods was then repeated on the downsampled data. As shown here, the number of variants called in the original data and downsampled data are comparable and, in every case, the downsampled data had a nearly 90% overlap with the variants called form the original data.

Supplemental Table S5. Number of variants detected with two different library preparations

Health Canada Mutamouse Animal	# Variants from Nextera Mate-Pair Library	# Variants from Illumina TruSeq Paired-End Library	Unique Variants from Nextera Mate-Pair Library	Unique Variants from Illumina TruSeq Paired-End Library	Intersection (%)
2	4,765,274	4,887,954	227,071	349,751	88.78

The original sequence data for Health Canada Mutamouse Animal 2 was generated from two different library preparations, namely Illumina TruSeq and Nextera Mate Pair libraries. This latter library preparation was used for the two Covance mice. To investigate the effect that the library preparation had on the variants called in this animal, the original BAM file was split based on read group to produce a BAM file for each library preparation method. The same analysis described in the Variant Calling section of the Methods was then repeated on the two newly produced BAM files. As shown here, the number of variants called from either library preparation is comparable and this split data had nearly 90% overlap in terms of the variants called.
